# Identification of Cellular Senescence-Related Hub Genes in Rheumatoid Arthritis from Bioinformatics Analysis Through Machine Learning up to Verifications in Mouse Macrophages and Tests in Patients

**DOI:** 10.3390/ijms27167493

**Published:** 2026-08-21

**Authors:** Dandan Wang, Linkun Tian, Qingshan Ma, Zhengdong Zhang, Yi Wang, Junhao Fang, Hairong Xu, Qi Chen, Hongdian Chen, Fangyuan Wang, Qiaoyan Zhang, Quanlong Zhang, Luping Qin

**Affiliations:** 1 School of Pharmaceutical Sciences, Zhejiang Chinese Medical University, Hangzhou 311402, China; 2 Zhejiang Key Laboratory of Chinese Medicine Modernization, Zhejiang Chinese Medical University, Hangzhou 311402, China

**Keywords:** rheumatoid arthritis, cellular senescence, RIPK2, macrophage, machine learning

## Abstract

Rheumatoid arthritis (RA) is a chronic autoimmune disease characterized by synovial inflammation and joint destruction. Cellular senescence contributes to chronic inflammation, yet key senescence-associated regulators in RA remain unclear. This study aimed to identify RA senescence-related signatures and their roles. Using GSE89408 as the training cohort, we screened hub genes by intersecting differentially expressed and senescence-related genes via WGCNA and three machine learning algorithms, with GSE55457 for external validation. Immune infiltration, regulatory network, subtyping and drug prediction were analyzed. Clinical and in vitro assays validated RIPK2 expression and function in the macrophage senescence-like phenotype, with preliminary signaling exploration. Three senescence-related hub genes (TNFAIP6, SLC2A3, RIPK2) were identified. The derived nomogram showed robust diagnostic performance (AUC = 0.988). Hub genes correlated strongly with myeloid cells, especially macrophages. Two immunologically distinct RA subtypes were identified. RIPK2 was upregulated in clinical samples; its inhibition attenuated LPS-induced macrophage senescence-like changes and inflammation. Preliminary data suggested RIPK2 may act via the NF-κB pathway. This study identifies RA senescence-associated signatures, revealing RIPK2 linking innate immunity to macrophage senescence-like changes, offering novel insights into pathogenesis and supporting it as a candidate biomarker and therapeutic target.

## 1. Introduction

Rheumatoid arthritis (RA) is a chronic autoimmune disease characterized by symmetric polyarticular erosive inflammation, with a global prevalence of approximately 0.5–1% [1]. It can lead to joint deformity, functional loss, and even disability, severely affecting patients’ quality of life and imposing a heavy socioeconomic burden [2]. The pathogenesis of RA is complex, involving genetic, environmental, and immune dysregulation factors, among which abnormal activation of immune cells and inflammatory factor storms are the core pathological features [3,4]. Although significant progress has been made in the diagnosis and treatment of RA in recent years, some patients still respond poorly to existing therapies, and specific biomarkers for early diagnosis are lacking. Therefore, in-depth exploration of key molecular targets in RA pathogenesis and the identification of new diagnostic and therapeutic biomarkers have important clinical significance [5].

Cellular senescence, characterized by stable cell-cycle arrest accompanied by metabolic alterations and the acquisition of a senescence-associated secretory phenotype (SASP), has emerged as an important biological process linking aging, chronic inflammation, and immune dysregulation [6,7]. Senescent cells are not merely passive markers of aging but can actively reshape tissue microenvironments through the secretion of pro-inflammatory cytokines, chemokines, and matrix-remodeling factors. Increasing evidence indicates that cellular senescence contributes to autoimmune and inflammatory diseases by sustaining chronic immune activation and tissue damage [8]. In RA, senescence-associated alterations have been observed in multiple cell populations, including synovial fibroblasts [9] and immune cells [10], suggesting that senescence-related mechanisms may participate in inflammatory amplification and joint destruction [11]. However, the key genes regulating cellular senescence in RA and the molecular mechanisms linking senescence to immune dysfunction remain largely unclear.

The rapid development of transcriptomic sequencing and computational biology provides powerful approaches for systematically identifying disease-associated molecular signatures from large-scale datasets. Integrative strategies combining differential expression analysis, network-based approaches, and machine learning have increasingly been applied to discover clinically relevant biomarkers and potential therapeutic targets [12]. However, cellular senescence-related molecular signatures in RA have not been comprehensively characterized, and their relationships with immune microenvironment remodeling remain poorly understood. In particular, whether senescence-associated molecular signatures are associated with specific immune phenotypes and contribute to inflammatory remodeling in RA remains incompletely understood.

In this study, we integrated transcriptomic analysis, cellular senescence-related gene profiling, weighted gene co-expression network analysis (WGCNA) [13], and machine learning approaches to identify senescence-associated hub genes involved in RA [14,15]. Comprehensive analyses of immune infiltration, regulatory networks, molecular subtypes, and potential therapeutic compounds were performed to characterize the biological functions and clinical relevance of these genes. Among the identified candidates, receptor-interacting serine/threonine kinase 2 (RIPK2), a key regulator of innate immune signaling, was selected for further experimental validation. We further investigated RIPK2 because it showed the most consistent performance across datasets. These findings provide new insights into the contribution of cellular senescence to RA pathogenesis and identify RIPK2 as a potential biomarker and therapeutic target for RA intervention.

## 2. Results

### 2.1. Study Design

**Figure 1:**
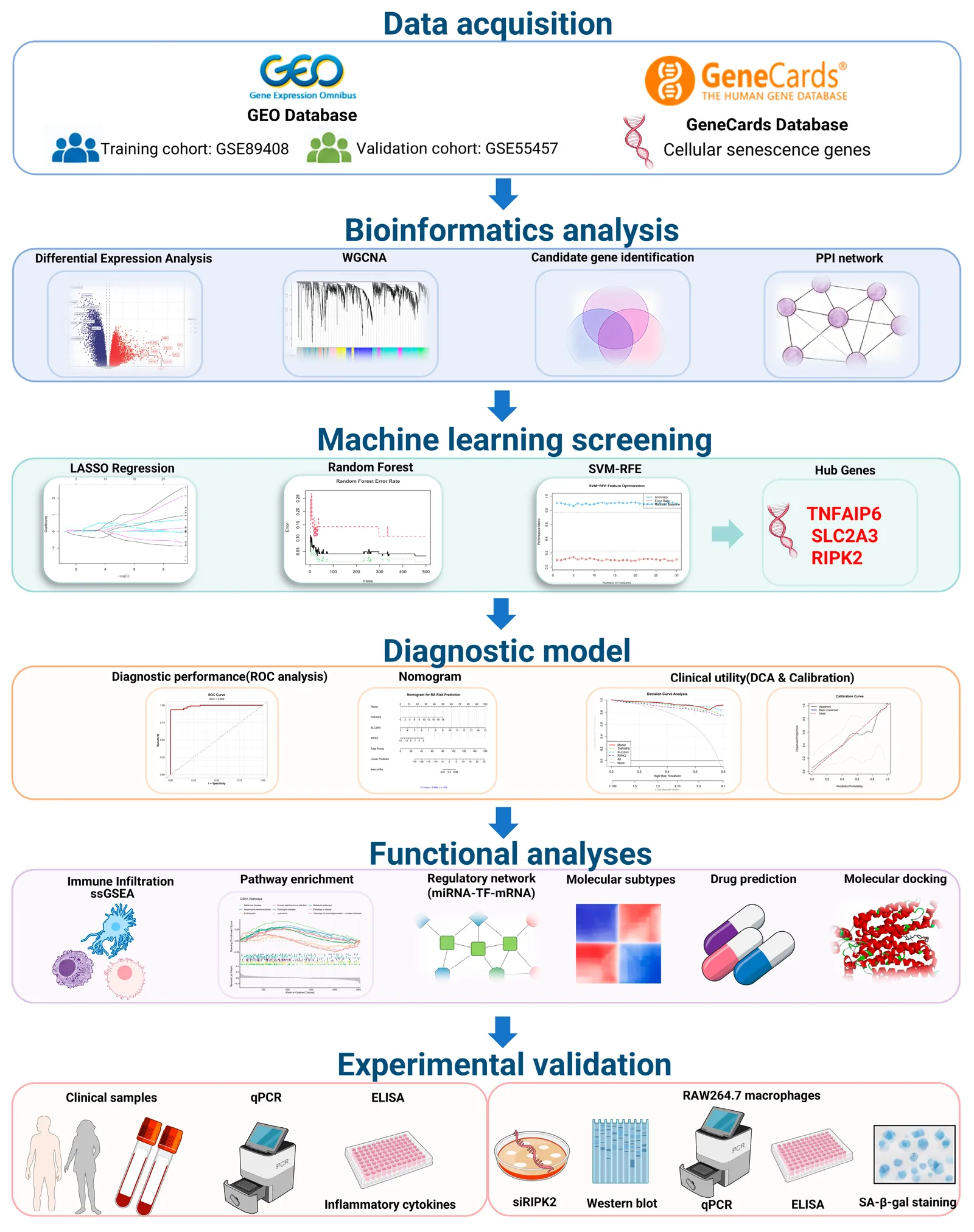
Workflow of the study design.

Figure 1 displays the full analytical workflow of our research. First, we retrieved gene expression datasets of RA synovial samples (GSE89408 and GSE55457) from the Gene Expression Omnibus (GEO) database (https://www.ncbi.nlm.nih.gov/geo/, accessed on 18 March 2026). These datasets contained a total of 218 synovial samples, including 152 rheumatoid arthritis (RA) patients, 22 osteoarthritis (OA) patients, 28 healthy controls, 10 patients with arthralgia, and 6 patients with undifferentiated arthritis. For subsequent bioinformatic analyses, we only selected 28 normal synovial tissues and 95 samples from patients with established RA. GSE55457 served as the independent external validation cohort.

### 2.2. Identification of Differentially Expressed Genes

**Figure 2:**
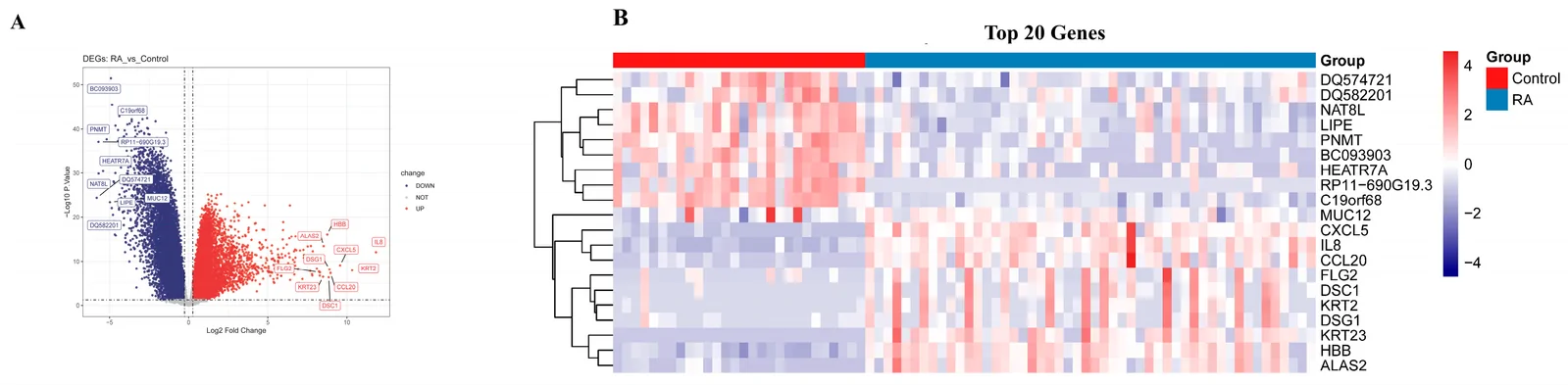
Differential gene expression between RA and control groups. (**A**) Red and blue represent DEGs with significantly higher or lower expression levels in the RA group, respectively. The vertical dashed lines indicate |log_2_FC| = 0.5, and the horizontal dashed line represents *p*adj = 0.05; grey dots indicate non-differentially expressed genes. (**B**) Heatmap showing the top 20 genes with significantly different expression between RA and control groups.

A total of 6585 DEGs were identified from the RA dataset, with *p*adj < 0.05 and |log_2_FC| > 0.5. The volcano plot and heatmap shown in Figure 2A,B demonstrate the differential expression patterns of these DEGs, respectively.

### 2.3. Weighted Gene Co-Expression Network Analysis (WGCNA) and Critical Module Identification

**Figure 3:**
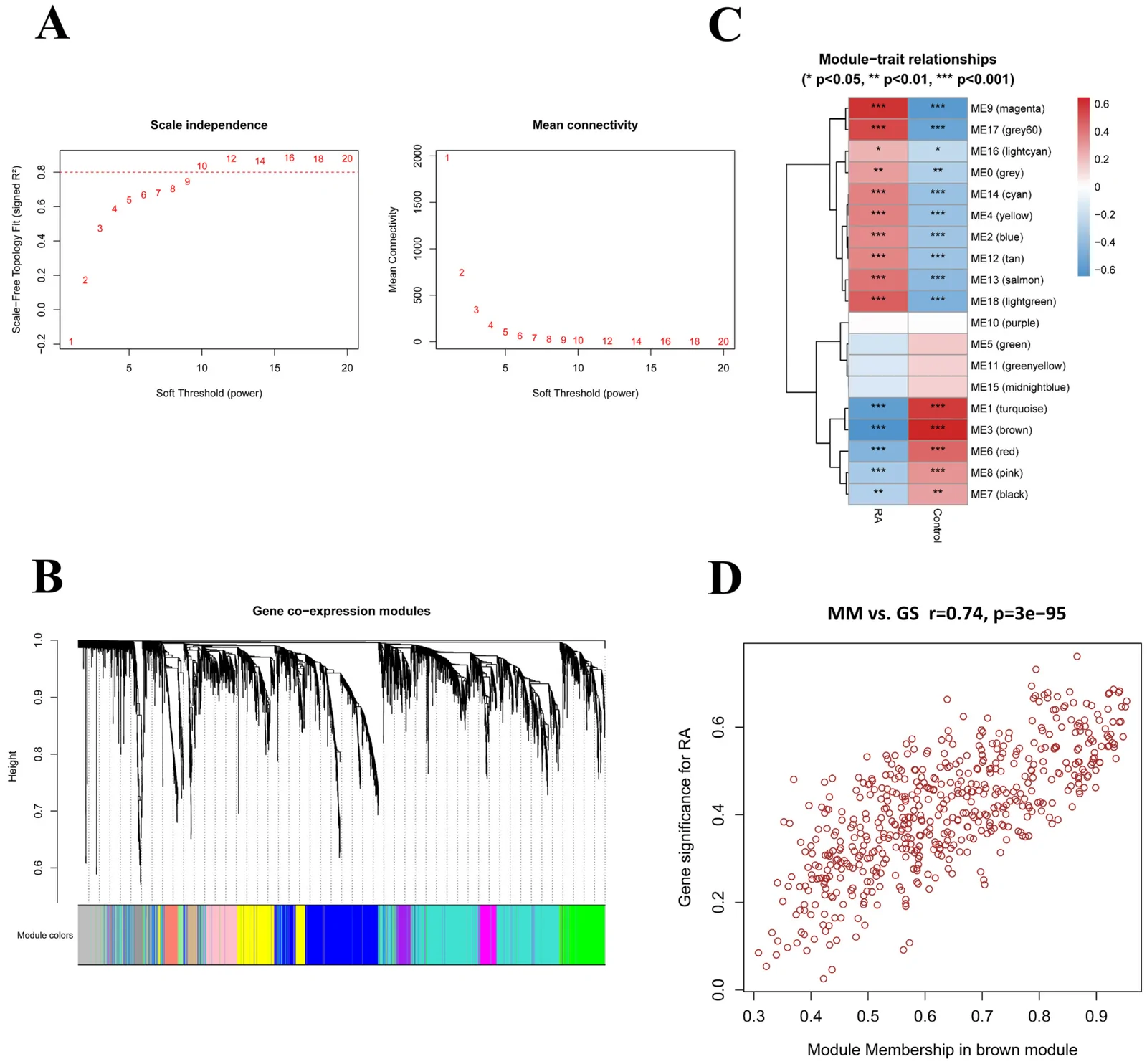
WGCNA co-expression network analysis identifies key gene modules associated with RA. (**A**): Selection of the soft-thresholding power. Left: scale-free topology fit index across different soft thresholds; the red dashed line denotes the reference threshold (fit index = 0.85) used to determine the optimal soft threshold power. Right: mean connectivity of the network across different soft thresholds. (**B**) Gene co-expression module clustering dendrogram. Genes are grouped into distinct co-expression modules via hierarchical clustering. Each module is marked with a unique color in the bottom annotation bar, and genes not assigned to any specific module are classified into the grey module. (**C**) Module–trait correlation heatmap, showing the correlation coefficients and statistical significance between each gene module and RA and control traits. (**D**) Scatter plot of module membership versus gene significance for the brown module.

We constructed a scale-free co-expression network using weighted gene co-expression network analysis to identify the most critical gene modules associated with RA. Based on the scale independence and mean connectivity of the network, we selected 10 as the soft threshold (Figure 3A). Hierarchical clustering based on the similarity of gene expression profiles divided all genes into multiple co-expression modules with different expression patterns, each identified by a different color (Figure 3B). Further correlation analysis between module eigengenes and clinical traits revealed that multiple modules were significantly associated with the RA phenotype, with the brown module containing 543 genes showing the strongest correlation with RA traits (Figure 3C). The Pearson product-moment correlation coefficient between module membership (MM) and gene significance (GS) revealed a significant correlation (r = 0.74, *p* = 3 × 10^−95^), calculated via a two-sided Student’s *t*-test with *n* − 2 degrees of freedom. This result indicates that genes within this module exhibit both high intra-module co-expression consistency and strong association with the RA phenotype, supporting the brown module as the key module for subsequent core target gene screening (Figure 3D).

### 2.4. Identification of Candidate Hub Genes via Machine Learning

**Figure 4:**
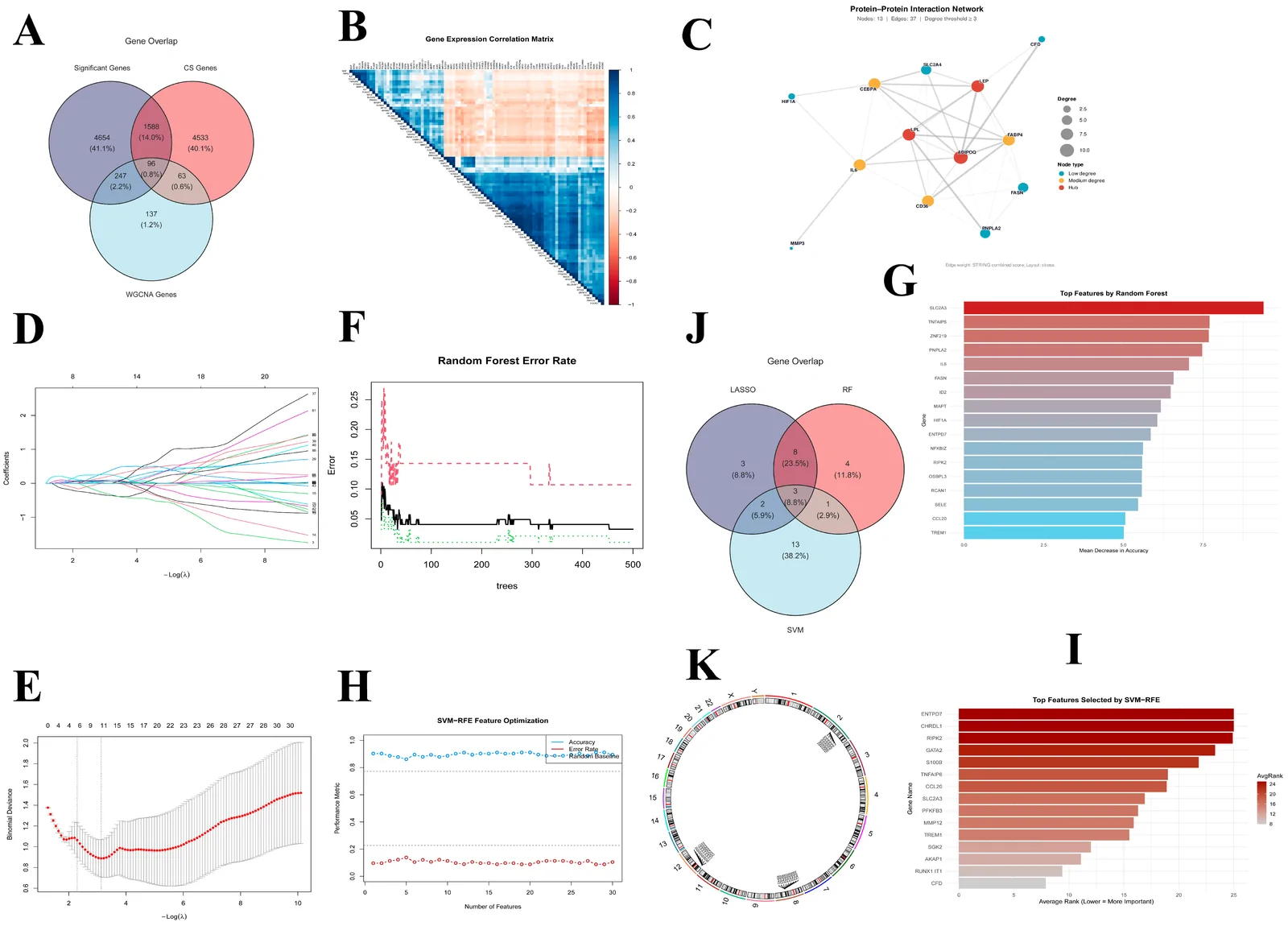
Identification of hub genes using integrated machine learning approaches in RA. (**A**) Venn diagram of DEGs, senescence related genes, and WGCNA module genes. (**B**) Correlation heatmap of candidate genes. (**C**) PPI network of candidate genes. (**D**,**E**) LASSO regression analysis for feature selection. (**F**) Error convergence curve of the random forest model. The black solid line indicates the overall out of bag (OOB) error rate, the red dashed line indicates the class specific OOB error rate of the RA group, and the green dotted line indicates the class specific OOB error rate of the control group. (**G**) Random forest feature importance ranked by mean decrease in accuracy. Bars are sorted in descending order of importance; the gradient color from red to blue is used only for visual enhancement and does not represent different gene groups. (**H**,**I**) SVM RFE analysis for optimal feature selection. (**J**) Intersection of three machine learning methods identifying hub genes. (**K**) Chromosomal distribution of hub genes.

To screen core diagnostic target genes for RA, this study integrated differentially expressed genes, cellular senescence-related genes, and WGCNA key module genes, and obtained 96 candidate genes by taking the intersection of the three (Figure 4A). Gene expression correlation analysis and protein–protein interaction network analysis showed close expression correlations and protein interactions among candidate genes (Figure 4B,C). Three machine learning methods were further employed for feature selection: LASSO regression screened feature genes with non-zero coefficients through regularization compression (Figure 4D,E); random forest evaluated gene importance based on Gini index decrease, with error rapidly converging as the number of decision trees increased (Figure 4F,G); and SVM-RFE recursive feature elimination screened the optimal feature subset, with model accuracy stably maintained at a high level of approximately 90% (Figure 4H,I). The feature genes screened by the three methods were intersected to obtain the final core diagnostic gene set (Figure 4J), with the final hub gene list being TNFAIP6, SLC2A3, and RIPK2; their chromosomal distribution is shown in Figure 4K. These results indicate that the core genes screened by cross-validation with multiple machine learning methods have reliable disease discrimination ability and can serve as key targets for RA diagnosis and mechanistic research.

### 2.5. Expression Validation and Diagnostic Performance of Hub Genes in Training and Validation Cohorts

To systematically evaluate the expression characteristics and diagnostic value of the three screened hub genes (TNFAIP6, SLC2A3, and RIPK2) in RA, we performed expression comparison and diagnostic efficacy assessment in both the training dataset (GSE89408) and an independent external validation dataset (GSE55457).

**Figure 5:**
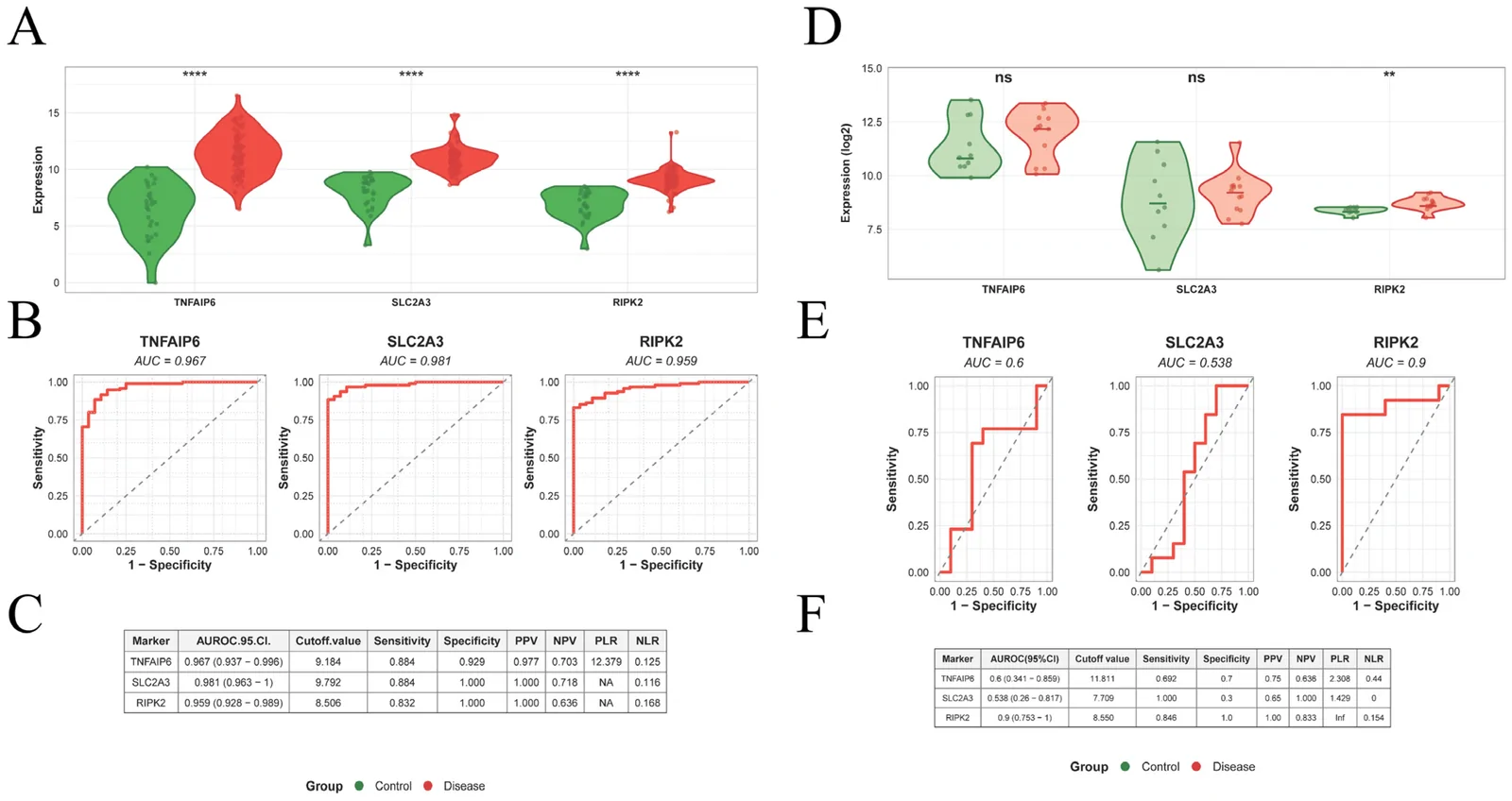
Expression and diagnostic efficacy validation of hub genes in training and external validation cohorts. (**A**) Violin plots of three hub genes in the training cohort (GSE89408). Green, control; red, RA. **** *p* < 0.0001. (**B**) ROC curves of individual hub genes in the training cohort. (**C**) Detailed diagnostic indicators of hub genes in the training cohort. (**D**) Violin plots of three hub genes in the validation cohort (GSE55457). Green, control; red, RA. ns, not significant; ** *p* < 0.01. (**E**) ROC curves of individual hub genes in the validation cohort. (**F**) Detailed diagnostic indicators of hub genes in the validation cohort.

In the training cohort, violin plot visualization revealed that the expression levels of all three hub genes were markedly elevated in RA samples relative to healthy controls, with all comparisons achieving extremely significant statistical differences (all *p* < 0.0001, Figure 5A). This consistent upregulation pattern indicates that these genes may play functional roles in the onset and progression of RA.

Subsequently, receiver operating characteristic (ROC) curves were constructed to quantify the diagnostic capacity of each hub gene. The area under the ROC curve (AUC) was 0.967 (95% CI: 0.937–0.996) for TNFAIP6, 0.981 (95% CI: 0.963–1.000) for SLC2A3, and 0.959 (95% CI: 0.928–0.989) for RIPK2, respectively (Figure 5B,C). All three genes exhibited excellent discriminative performance for distinguishing RA patients from healthy individuals. Further analysis of diagnostic metrics at the optimal cutoff values confirmed high sensitivity and specificity for all markers.

For the external validation using GSE55457, we replicated the analytical workflow to verify the robustness of our findings. The expression of TNFAIP6 and SLC2A3 showed a general upward trend in the RA group but did not reach statistical significance (both *p* > 0.05), whereas RIPK2 remained significantly upregulated in RA samples compared with controls (*p* < 0.01, Figure 5D). In terms of diagnostic efficacy, RIPK2 maintained favorable discriminative ability in the validation cohort, with an AUC of 0.900 (95% CI: 0.753–1.000). In contrast, TNFAIP6 and SLC2A3 showed moderate diagnostic performance in the validation set, with AUC values of 0.600 (95% CI: 0.341–0.859) and 0.538 (95% CI: 0.260–0.817), respectively (Figure 5E,F). Among the three candidate genes, RIPK2 showed the most consistent expression pattern across the external validation datasets, whereas the reproducibility of TNFAIP6 and SLC2A3 was limited.

In summary, all three hub genes are significantly upregulated in RA and present outstanding diagnostic efficacy in the training cohort. Among them, RIPK2 demonstrates the most stable diagnostic performance across independent datasets, supporting its potential as a reliable diagnostic biomarker for RA.

### 2.6. Diagnosis Value Evaluation

**Figure 6:**
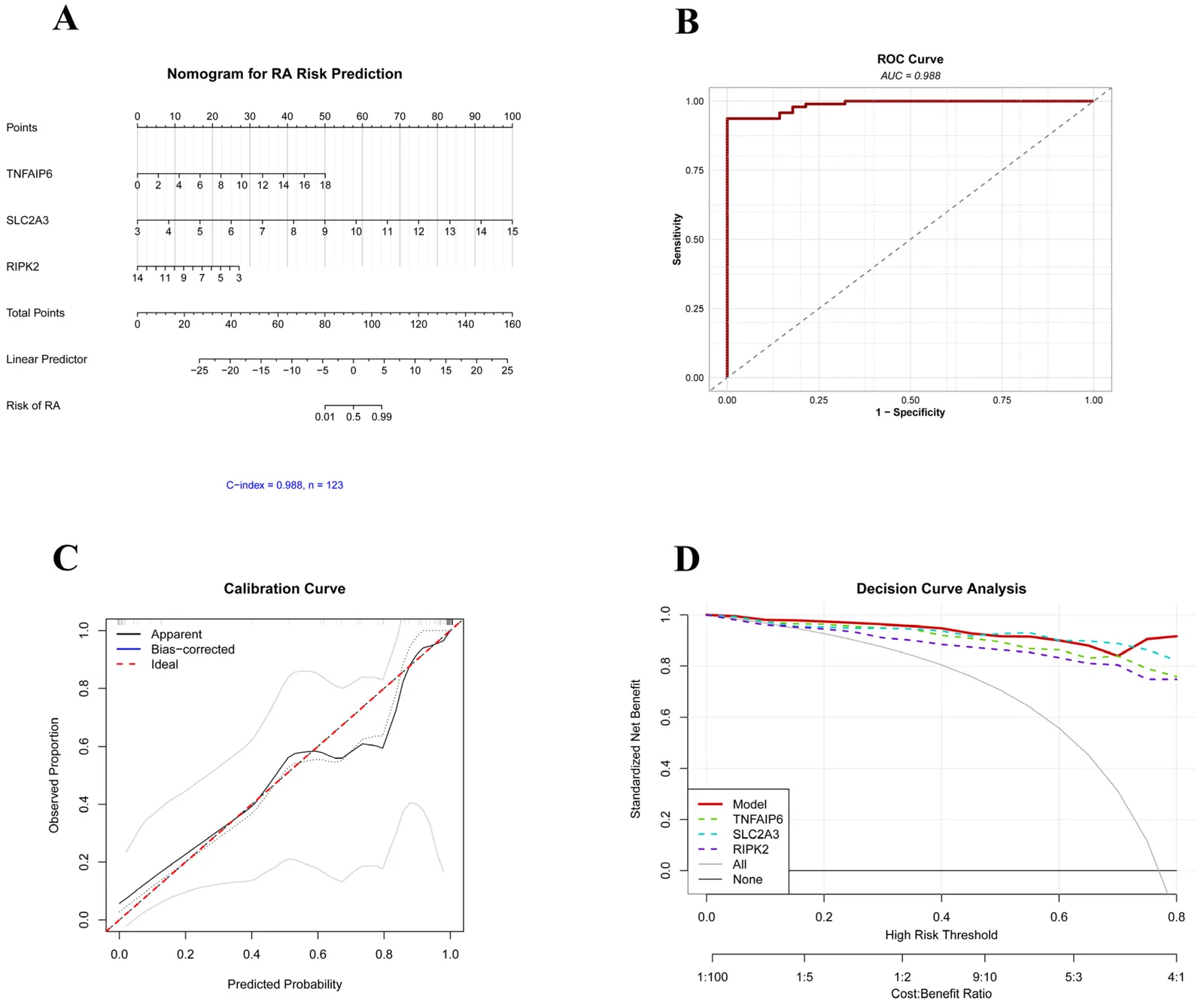
Construction and evaluation of a nomogram model based on hub genes for RA. (**A**) Nomogram for RA risk prediction based on TNFAIP6, SLC2A3, and RIPK2. (**B**) ROC curve of the nomogram model. (**C**) Calibration curve of the prediction model. The red dashed line (Ideal) represents the perfect calibration reference, in which predicted probability fully matches the observed proportion. The black solid line indicates apparent calibration performance, the blue solid line represents the bias-corrected calibration curve derived from bootstrap resampling, and the grey lines denote the upper and lower boundaries of the 95% confidence interval for the bias-corrected curve. (**D**) Decision curve analysis showing clinical utility.

Based on the three screened core genes (TNFAIP6, SLC2A3, RIPK2), a nomogram prediction model for RA risk was constructed. The nomogram assigns corresponding scores according to the expression level of each gene, and the individual scores of the three genes are summed to obtain a total score, which can be converted to the probability of developing RA through the conversion between total score and linear predictor value, enabling intuitive calculation of individual RA risk (Figure 6A). ROC curve evaluation of the model’s diagnostic efficacy showed that the area under the curve (AUC) reached 0.988, indicating that this nomogram model has extremely high discrimination and diagnostic accuracy for RA (Figure 6B). Calibration curves further validated the model’s prediction accuracy, with both the apparent curve and bias-corrected curve fitting well with the ideal diagonal line, suggesting high consistency between model-predicted values and actual observed values (Figure 6C). Decision curve analysis (DCA) was employed to assess the clinical utility of the nomogram. The results indicated that, across a wide spectrum of high-risk thresholds, the standardized net benefit of the nomogram consistently surpassed that of single-gene predictors alone, underscoring its robust clinical applicability and its value in informing treatment decisions (Figure 6D). In summary, the nomogram developed from the three core genes not only exhibits robust diagnostic performance but also offers tangible clinical benefits, thereby serving as a practical visual tool for individualized RA risk stratification.

### 2.7. Correlation Between the Immune Cell Infiltration and Hub Genes

To systematically characterize the immune microenvironment features of RA and the immune regulatory mechanisms of core genes, this study employed ssGSEA to evaluate the infiltration levels of 28 immune cell types, combined with correlation analysis and GSVA enrichment analysis to verify the immune regulatory roles of core genes.

**Figure 7:**
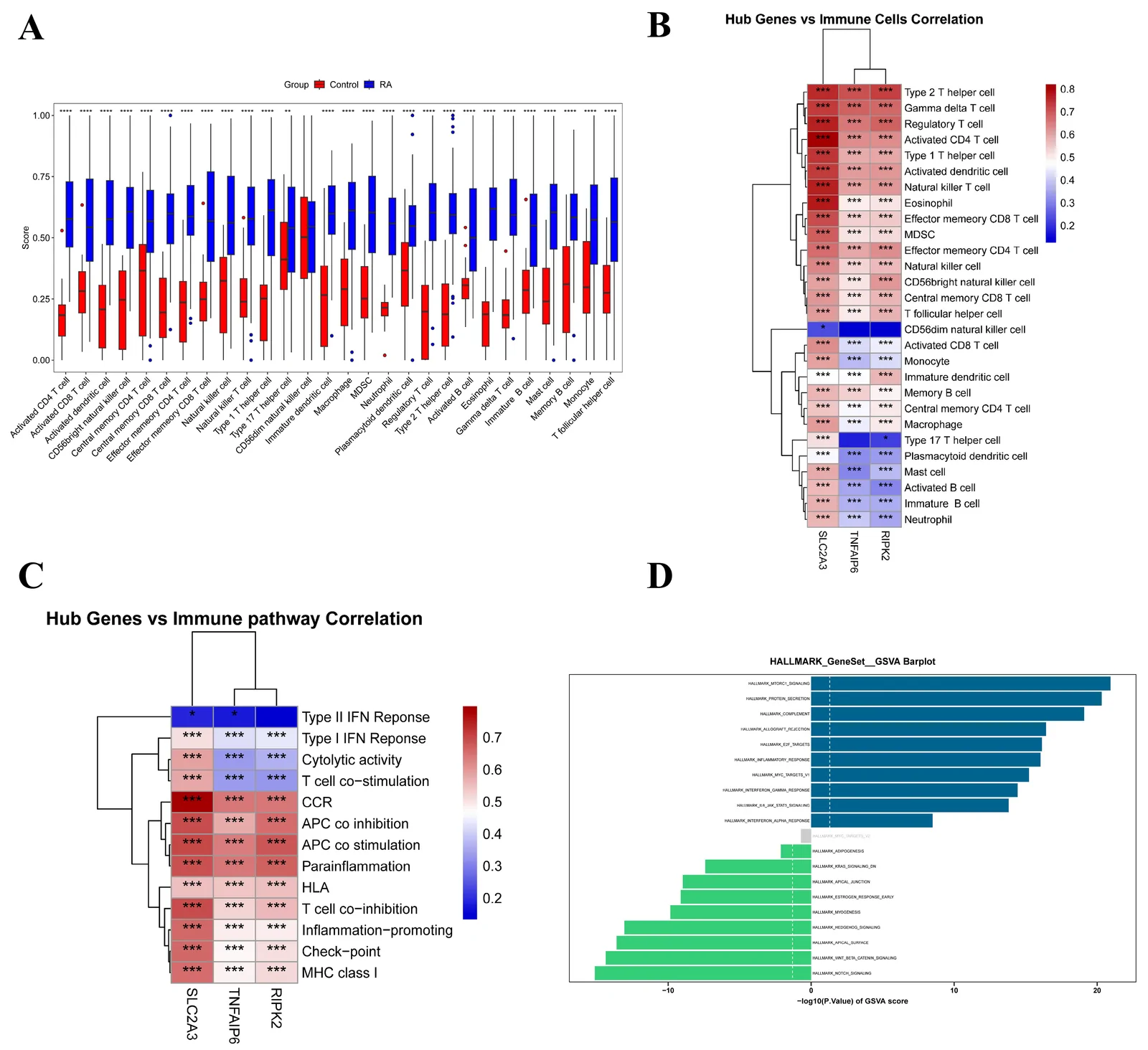
Immune infiltration landscape and correlation analysis of hub genes in RA. (**A**) ssGSEA scores of 28 immune cell types. (**B**) Correlation between hub genes and immune cell infiltration. (**C**) Correlation between hub genes and immune-related pathways. (**D**) GSVA enrichment of HALLMARK pathways. Blue bars indicate pathways significantly up-regulated in RA, while green bars indicate pathways significantly down-regulated in RA. The two vertical dashed lines in the blue and green regions denote the statistical significance threshold corresponding to *p* = 0.05. Bars extending outward beyond the dashed lines represent pathways with statistically significant enrichment. * *p* < 0.05, ** *p* < 0.01, *** *p* < 0.001, **** *p* < 0.0001.

The results showed that compared with the control group, the enrichment scores of multiple immune cell subsets were significantly increased in the RA group, including activated CD4+/CD8+ T cells, type 1/type 17 helper T cells, macrophages, monocytes, neutrophils and natural killer cells, suggesting that the immune microenvironment of RA patients is in a state of widespread immune activation (Figure 7A). All three hub genes showed extremely significant positive correlations with the majority of immune cell subsets, among which T cell subsets exhibited relatively higher correlation coefficients. Meanwhile, myeloid innate immune cells including macrophages, monocytes and dendritic cells also presented consistently extremely significant positive correlations with all three hub genes. Combined with the innate immune regulatory function of hub genes and the core pathological features of RA synovial inflammation, these findings suggest that the core genes may participate in the pathogenesis of RA mainly by regulating the activation of innate immune cells (Figure 7B). Immune pathway correlation analysis further verified that core genes were significantly positively correlated with pathways including CCR, APC co-stimulation/co-inhibition, parainflammation, HLA, T cell co-inhibition, inflammation promotion, checkpoints, and MHC class I (all *p* < 0.001), while correlations with type I/type II IFN response, cytolytic activity, and T cell co-stimulation pathways were relatively weaker (Figure 7C). GSVA enrichment analysis showed that multiple HALLMARK characteristic gene sets were significantly differentially enriched in RA, including classical inflammatory pathways such as inflammatory response, interferon response and IL-6/JAK/STAT3 signaling, further supporting the widespread activation of immune-inflammatory pathways in RA (Figure 7D).

Furthermore, immune cell infiltration heatmaps and immune cell correlation matrix analysis showed obvious differences in immune infiltration profiles between the RA and control groups, and extensive positive correlations existed among various immune cell subsets (Supplementary Figure S1A,C); inter-group comparisons of immune pathway enrichment scores and GSVA enrichment analysis of C7 immune-related gene sets and KEGG pathways further verified the abnormal activation of immune-inflammatory pathways in RA from multiple dimensions (Supplementary Figure S1B,D–F).

In summary, core genes are closely related to various immune cells and inflammatory pathways in the RA immune microenvironment, and may participate in the pathogenesis of RA by regulating the interaction between innate and adaptive immunity.

### 2.8. Identification of Regulatory Signatures

**Figure 8:**
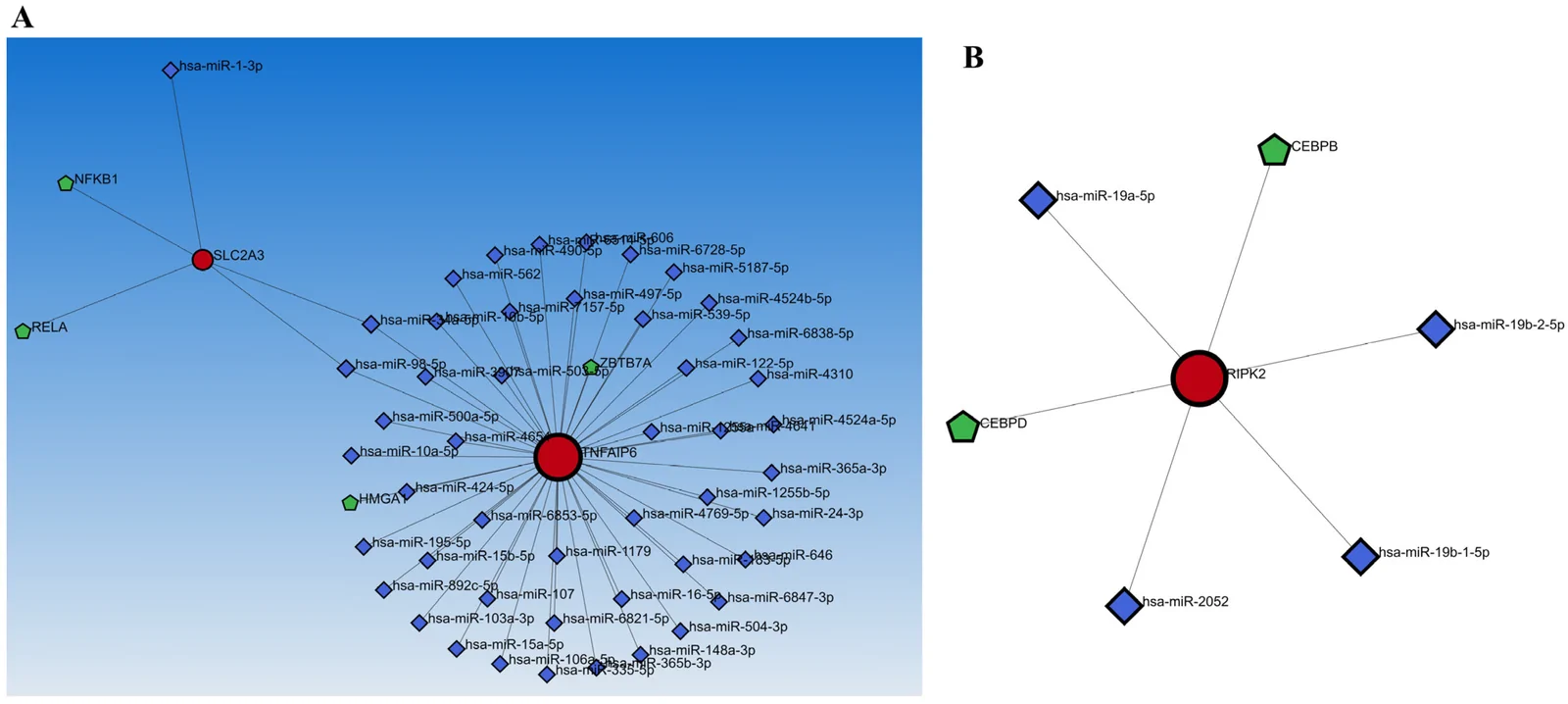
miRNA and transcription factor regulatory networks of hub genes in RA. (**A**) miRNA–TF–gene regulatory network for TNFAIP6 and SLC2A3. (**B**) miRNA–TF regulatory network of RIPK2. Red large circles represent hub target genes; blue diamonds represent microRNAs; green pentagons represent transcription factors. Lines between nodes indicate predicted regulatory interactions among molecules.

To systematically characterize the upstream transcriptional and post-transcriptional regulatory mechanisms of core genes, this study constructed miRNA and transcription factor (TF) regulatory networks for core genes. The results showed that both SLC2A3 and TNFAIP6 are targeted by a large number of miRNAs and are closely associated with key inflammatory transcription factors such as NFKB1 and RELA. Further analysis of RIPK2 revealed that it is dually and synergistically regulated by miRNAs and transcription factors: miRNAs such as hsa-miR-19a-5p, hsa-miR-19b-1-5p, hsa-miR-19b-2-5p, and hsa-miR-2052 can regulate RIPK2 expression at the post-transcriptional level, while transcription factors such as CEBPB and CEBPD participate in its expression regulation at the transcriptional level, together constituting a complex regulatory network (Figure 8A,B). These results indicate that all three core genes are subject to multi-level regulation by miRNAs and transcription factors, providing new perspectives for in-depth understanding of the molecular regulatory mechanisms of RA and the development of targeted therapeutic strategies.

### 2.9. Identification of Hub Subtypes

**Figure 9:**
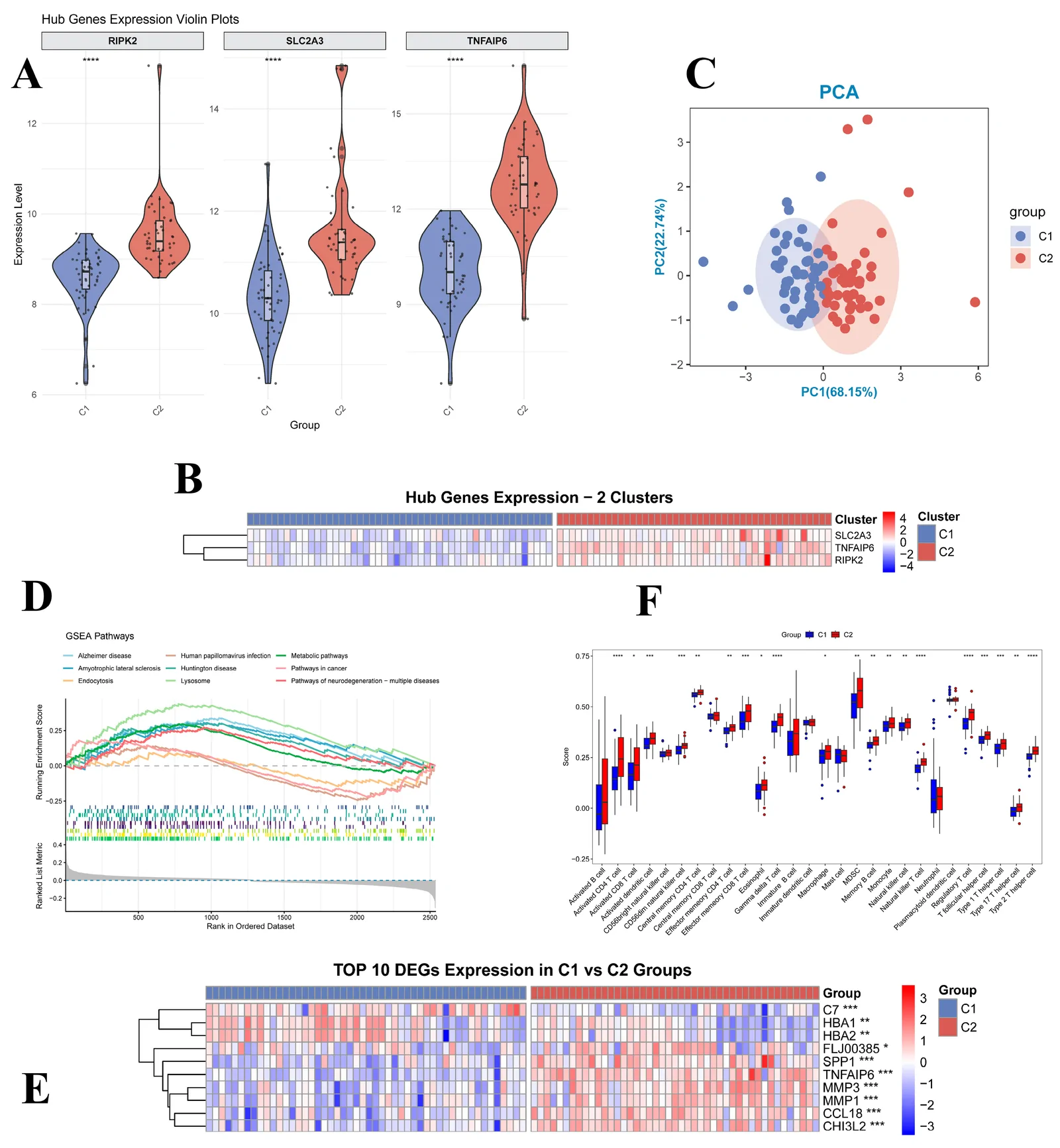
Molecular subtype analysis of RA based on hub gene expression. (**A**) Expression of hub genes in two molecular subtypes. (**B**) Heatmap of subtype-specific expression patterns. (**C**) PCA showing separation of two subtypes. (**D**) GSEA enrichment analysis between subtypes. The upper grey horizontal dashed line is the reference line at running enrichment score = 0 for distinguishing positive and negative enrichment. The lower blue horizontal dashed line represents the baseline of ranked list metric = 0. The grey area denotes the distribution range of the ranked list metric. Colored lines correspond to running enrichment curves of different gene sets; vertical tick marks at the bottom indicate the positions of gene-set members in the ordered dataset. (**E**) Differentially expressed genes between subtypes. (**F**) Immune infiltration differences between subtypes. * *p* < 0.05, ** *p* < 0.01, *** *p* < 0.001, **** *p* < 0.0001.

To explore the heterogeneity of RA mediated by core genes, this study performed consensus clustering based on the expression profiles of three core genes, dividing RA patients into two molecular subtypes (C1 and C2). Both violin plots and heatmaps showed that the expression levels of the three core genes were significantly higher in the C2 subtype than in the C1 subtype (all *p* < 0.0001), suggesting obvious gene expression heterogeneity between the two subtypes (Figure 9A,B). Principal component analysis (PCA) further verified that the expression profiles based on core genes can effectively distinguish the two RA subtypes, indicating that core genes have good subtype classification ability (Figure 9C). GSEA enrichment analysis showed significant enrichment differences between the two subtypes in metabolic pathways, cancer pathways and neurodegenerative disease pathways (Figure 9D). Differential expression analysis screened the top 10 differentially expressed genes between the two subtypes, including C7, HBA1, HBA2, FLJ00385, SPP1, TNFAIP6, and MMP3, whose expression patterns showed significant differences between the two subtypes (Figure 9E). Immune infiltration analysis indicated that ssGSEA enrichment scores of various immune cells and immune pathways were generally higher in the C2 subtype than in the C1 subtype, suggesting that the C2 subtype has a stronger immune activation phenotype (Figure 9F). In summary, RA can be divided into two subtypes with different molecular characteristics and immune phenotypes based on core genes, providing a theoretical basis for precise subtyping and individualized treatment of RA.

### 2.10. Discovery of Potential Small Molecules

**Figure 10:**
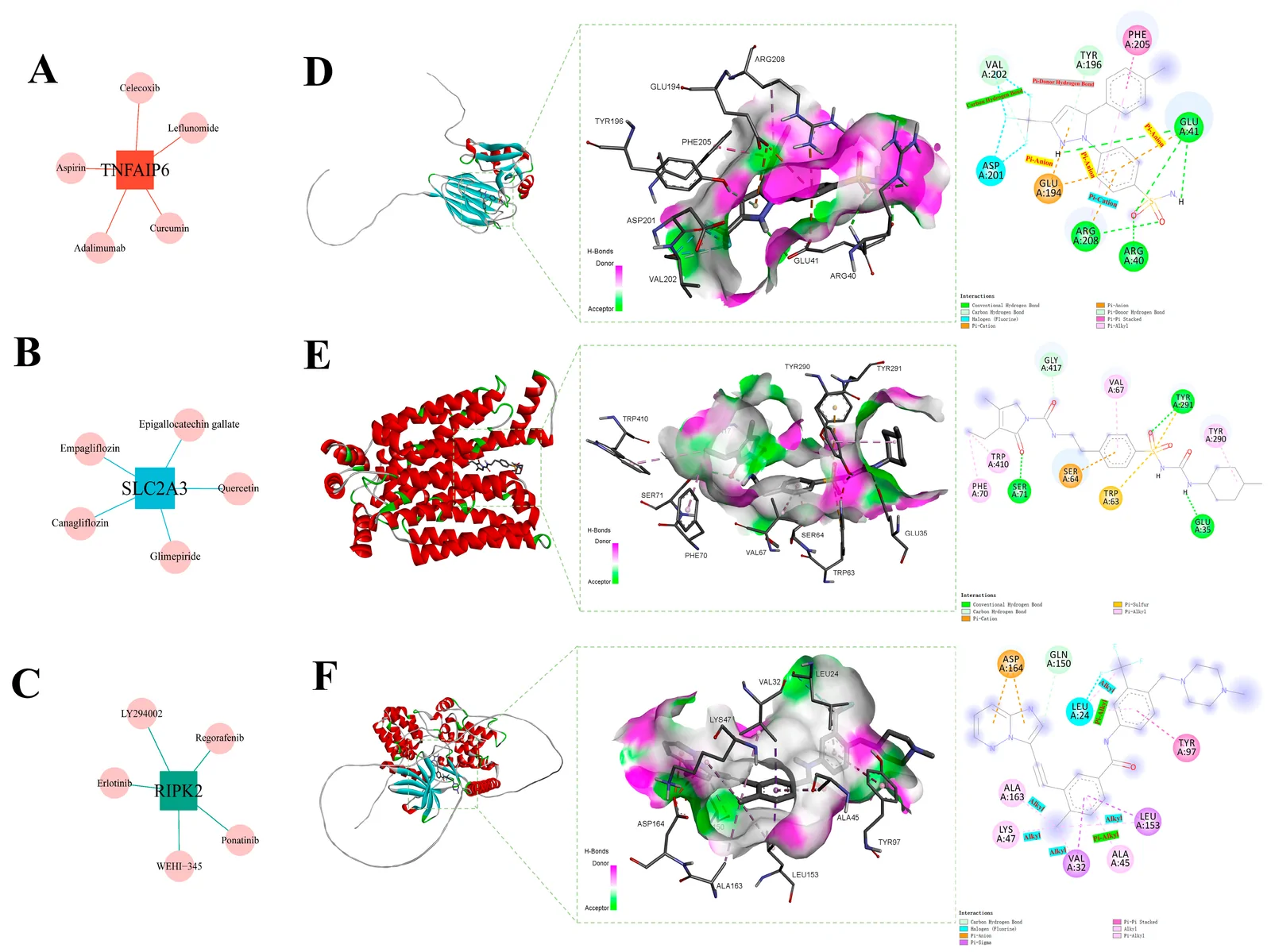
Identification of candidate drugs targeting hub genes and molecular docking validation. (**A**–**C**) Drug–gene interaction networks for TNFAIP6, SLC2A3, and RIPK2. (**D**–**F**) Representative molecular docking results of selected drug–protein complexes. In the docking structures, red ribbons represent α-helices, cyan ribbons represent β-sheets, grey lines represent disordered polypeptide regions, and green segments indicate loop regions. The small-molecule ligand is shown as stick model within the binding pocket. Manual text annotations have been added onto the two dimensional ligand receptor interaction diagrams (**D**,**F**) for visually confusable interaction types to improve readability.

Through drug–gene interaction network analysis, potential targeted drugs for the three core genes were screened (Figure 10A–C): TNFAIP6 can bind to various anti-inflammatory drugs and natural active ingredients such as aspirin, celecoxib, leflunomide, and curcumin; candidate drugs for SLC2A3 include glimepiride, quercetin, etc.; and RIPK2 interacts with kinase inhibitors such as ponatinib and erlotinib.

To further verify the binding ability of core genes to potential targeted drugs, this study selected three representative drug–protein combinations for molecular docking validation (Figure 10D–F). The results showed that the docking binding energy of celecoxib and P98066_TNFAIP6_HUMAN was −8.9 kcal/mol. Celecoxib can form stable binding with the active pocket of TNFAIP6 protein (P98066), forming a stable complex conformation through various intermolecular forces such as hydrogen bonds and hydrophobic interactions; the docking binding energy of glimepiride and SLC2A3_4ZW9 was −10.5 kcal/mol. The docking results of glimepiride and SLC2A3 protein (PDB ID: 4ZW9) showed that the small drug molecule can embed into the substrate-binding pocket of the protein and form close interactions with key amino acid residues; the docking binding energy of ponatinib and O43353_RIPK2_HUMAN was −10.1 kcal/mol. Molecular docking of ponatinib and RIPK2 protein (O43353) also showed good binding affinity, with the drug molecule forming stable binding with the ATP-binding pocket of the kinase domain. These molecular docking results verify the binding feasibility of core gene-encoded proteins and potential targeted drugs from a structural biology perspective, providing a theoretical basis for subsequent experimental validation and drug development.

### 2.11. Experimental Validation of RIPK2 Expression and Cellular Senescence Phenotype in RA

**Figure 11:**
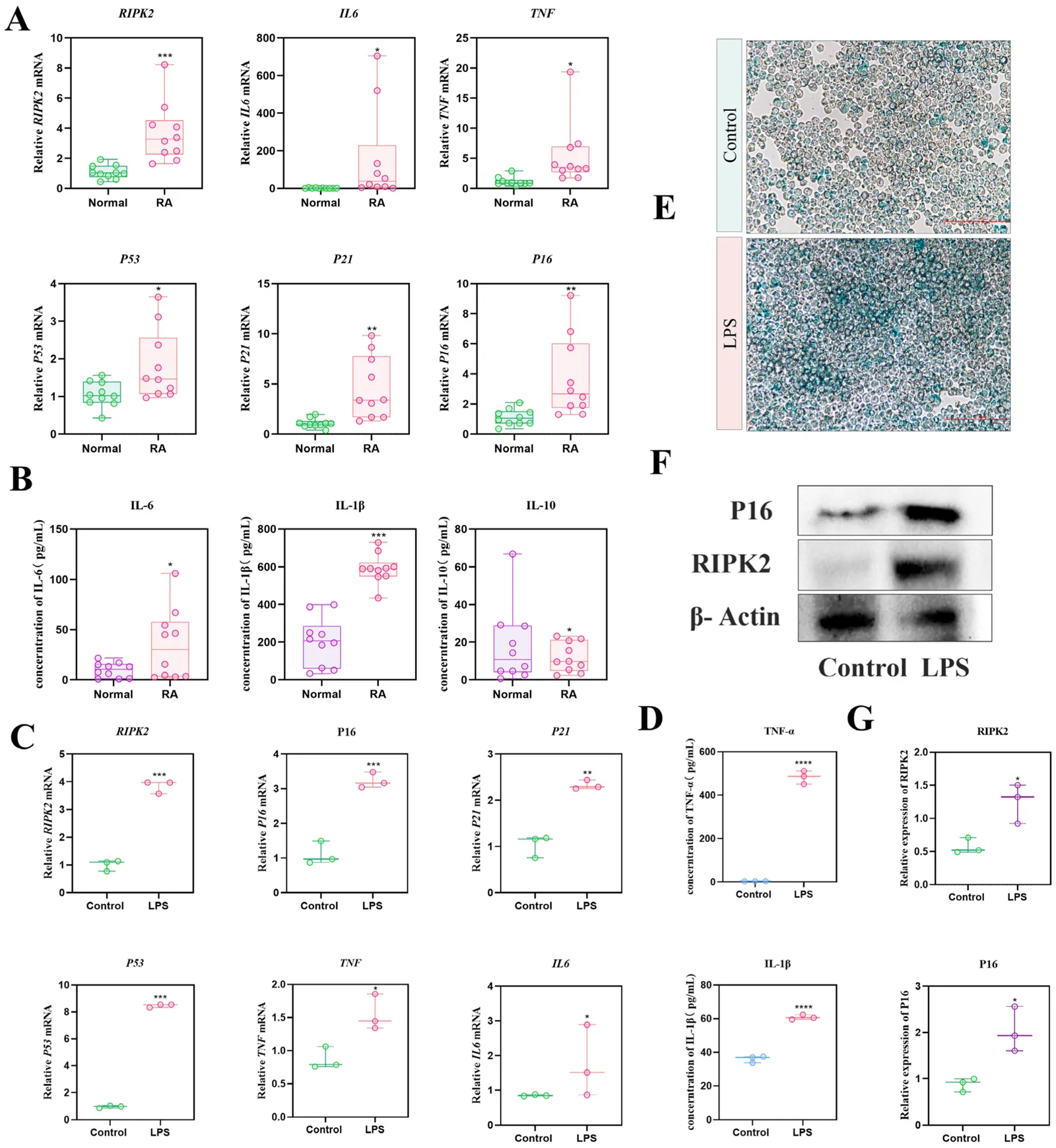
Experimental validation of RIPK2 expression and cellular senescence-like phenotype. (**A**) mRNA expression levels of RIPK2 and senescence-related genes in peripheral blood leukocytes from RA patients (*n* = 10) and healthy controls (*n* = 10). (**B**) Comparison of plasma inflammatory factor concentrations between RA patients and healthy controls. (**C**) mRNA expression levels of RIPK2 and senescence-related genes in RAW264.7 cells. (**D**) Expression levels of inflammatory factors in cell supernatant. (**E**) SA-β-gal staining of RAW264.7 cells. (**F**,**G**) Western blot detection and quantitative analysis of RIPK2 and senescence-related proteins in RAW264.7 cells. Data in (**A**,**B**) are presented as box plots with individual data points, where boxes represent the median and interquartile range (IQR), whiskers extend to 1.5× IQR, and individual dots represent independent patient samples (*n* = 10 per group). Data in (**C**,**D**,**F**,**G**) are presented as mean ± SD from three independent biological replicates (*n* = 3). * *p* < 0.05, ** *p* < 0.01, *** *p* < 0.001, **** *p* < 0.0001.

The aforementioned bioinformatics analysis screened three core genes: TNFAIP6, SLC2A3, and RIPK2. Comprehensive analysis of each gene’s diagnostic efficacy, correlation with immune senescence pathways, and network centrality revealed that RIPK2 performed most prominently among the three. Moreover, its regulatory role in RA macrophage senescence has not been systematically reported. Therefore, this study selected RIPK2 as the key research object for experimental validation at both clinical sample and cellular levels. First, peripheral blood samples from RA patients and healthy controls were collected for clinical validation. Briefly, the RA cohort consisted of 10 patients (mean age ± SD, 61.50 ± 11.10 years; 60% female) with a mean disease duration of 4.90 ± 1.79 years. Mean DAS28-ESR was 3.57 ± 0.91, mean CRP was 19.72 ± 21.74 mg/L, and mean ESR was 38.30 ± 30.59 mm/h. Rheumatoid factor was 259.85 ± 375.49 IU/mL, and anti-CCP titer was 212.66 ± 212.83 U/mL. Glucocorticoids were used in 60% of patients, conventional synthetic DMARDs in 90%, and biologic or targeted DMARDs in 20%. Healthy controls were age- and sex-matched (*n* = 10). Detailed characteristics are provided in Supplementary Table S1. qPCR results showed that compared with the healthy control group, the mRNA expression level of RIPK2 in peripheral blood leukocytes of RA patients was significantly increased (Figure 11A); plasma inflammatory factor detection further confirmed a significant inflammatory activation state in RA patients, and RIPK2 expression showed a positive correlation trend with inflammatory factor levels (Figure 11B), suggesting that RIPK2 may be involved in the inflammatory pathological process of RA.

To further explore the regulatory effect of RIPK2 on cellular senescence, in vitro functional validation was performed in RAW264.7 macrophages. qPCR results showed that the expression levels of senescence-related genes in cells were significantly changed after RIPK2 intervention (Figure 11C); cell supernatant ELISA detection showed that the secretion levels of TNF-α and IL-1β were significantly increased after LPS treatment, further verifying the inflammatory activation state (Figure 11D); and β-galactosidase staining results showed that the proportion of senescent positive cells was significantly increased in the intervention group, suggesting that RIPK2 can regulate the macrophage senescence-like phenotype (Figure 11E). Western blot experiments further verified the expression changes in RIPK2 and senescence-related proteins at the protein level, and grayscale quantitative analysis showed statistically significant differences (Figure 11F,G). These experimental results mutually corroborate the bioinformatics analysis conclusions, indicating that RIPK2 may participate in the inflammatory pathological process of RA by regulating macrophage senescence.

### 2.12. RIPK2 Mediates LPS-Induced Macrophage Senescence-like Phenotype and Inflammatory Response

To investigate the regulatory role of RIPK2 on macrophage senescence-like phenotype and inflammatory response, this study first designed and screened three siRNA sequences targeting RIPK2. Western blot results showed that all three siRNAs could effectively knock down RIPK2 protein expression, among which siRIPK2-1 showed the best knockdown efficiency and was selected for subsequent functional experiments (Supplementary Figure S2).

**Figure 12:**
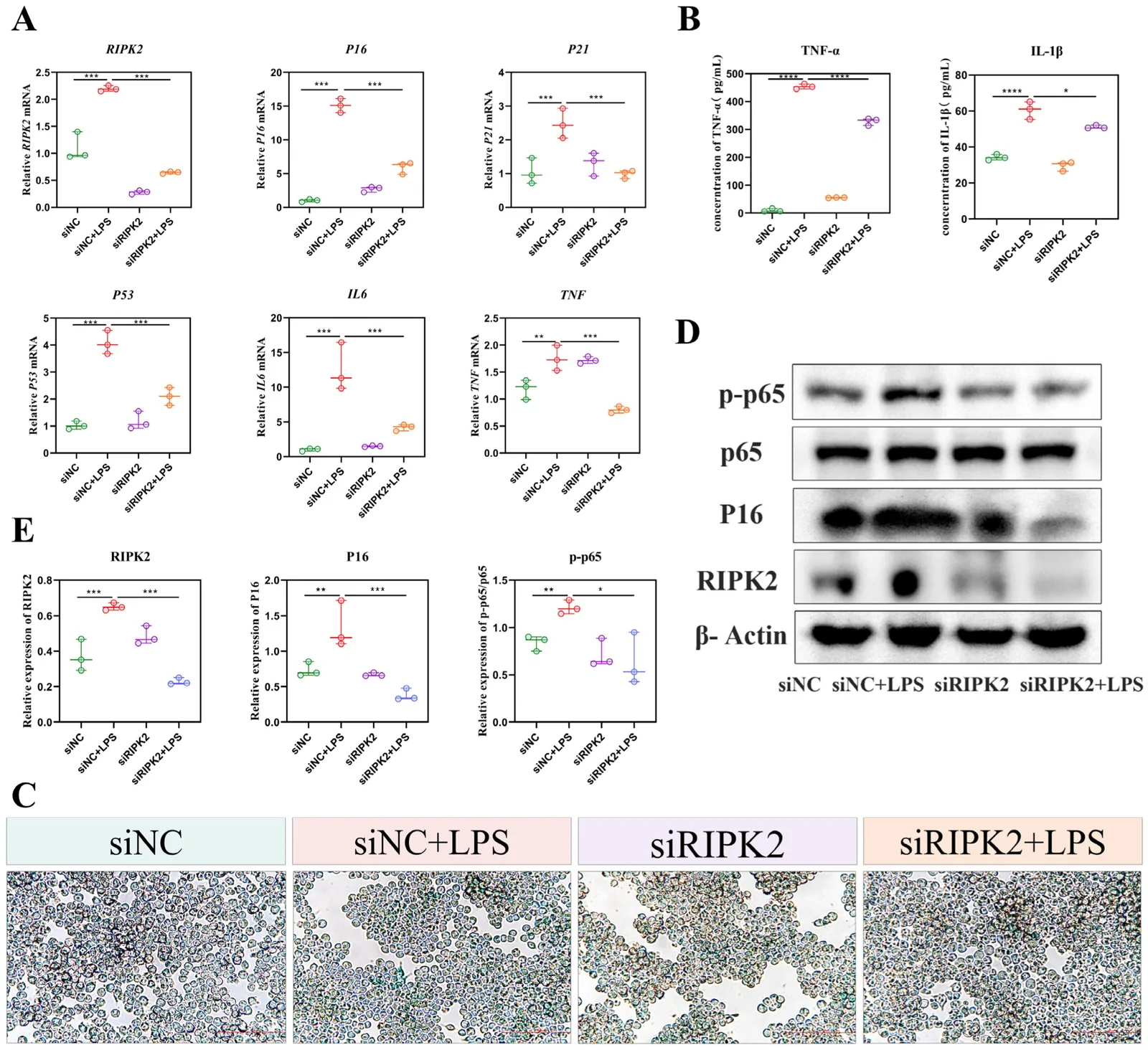
RIPK2 regulates LPS-induced macrophage senescence-like phenotype and inflammatory responses. (**A**) qPCR analysis of senescence and inflammatory genes. (**B**) Cytokine secretion levels. (**C**) SA-β-gal staining. (**D**,**E**) Western blot analysis. * *p* < 0.05, ** *p* < 0.01, *** *p* < 0.001, **** *p* < 0.0001.

Further validation of RIPK2 function was performed in an LPS-induced macrophage inflammation model. qPCR results showed that LPS stimulation significantly upregulated the mRNA expression of RIPK2, senescence-related genes (P16, P21, P53), and inflammatory factors (IL-6, TNF-α), while RIPK2 knockdown significantly inhibited the LPS-induced upregulation of senescence-related genes and inflammatory factors (Figure 12A). The secretion levels of TNF-α and IL-1β were significantly increased after LPS treatment, while RIPK2 knockdown significantly inhibited the LPS-induced secretion of inflammatory factors (Figure 12B). β-galactosidase staining results showed that LPS treatment significantly increased the proportion of senescent-positive cells, while RIPK2 knockdown significantly reversed the LPS-induced cellular senescence-like phenotype (Figure 12C). Western blot experiments further verified the above results at the protein level: LPS stimulation significantly increased the protein levels of RIPK2, P16, and phosphorylated p65 (p-p65), while RIPK2 knockdown effectively inhibited the LPS-induced upregulation of these proteins (Figure 12D,E). Notably, the LPS-induced increase in p-p65 was markedly attenuated by RIPK2 knockdown, suggesting that RIPK2 may regulate macrophage senescence-like changes and inflammatory responses at least in part through modulation of the NF-κB signaling pathway. In summary, RIPK2 is involved in regulating LPS-induced macrophage senescence-like phenotype and inflammatory response, and RIPK2 knockdown can alleviate the senescence-like phenotype and inflammatory activation state of macrophages, possibly via suppression of NF-κB activation.

## 3. Discussion

### 3.1. Identification of Senescence-Related Hub Genes in RA

RA is increasingly recognized as a disease driven not only by aberrant immune activation but also by age-related cellular dysfunction [16]. In the present study, we integrated transcriptomic profiling, cellular senescence-associated gene analysis, WGCNA, and three complementary machine learning algorithms to identify senescence-related molecular signatures in RA. Three hub genes (TNFAIP6, SLC2A3, and RIPK2) were consistently identified across multiple analytical approaches. Among these candidates, RIPK2 demonstrated the most stable diagnostic performance in an independent validation cohort and was therefore selected for experimental validation. Functional experiments further showed that RIPK2 knockdown attenuated LPS-induced macrophage senescence-like phenotype and inflammatory responses, supporting a potential role of RIPK2 in senescence-associated immune dysregulation in RA.

Growing evidence suggests that cellular senescence actively contributes to chronic inflammatory diseases through the SASP [17,18], which amplifies inflammatory signaling and reshapes the tissue microenvironment [19]. Rather than serving solely as a marker of aging, senescent immune and stromal cells are increasingly recognized as active participants in RA pathogenesis [20,21].

### 3.2. RIPK2 as a Candidate Regulator of Macrophage Senescence-like Phenotype

Among the three hub genes, RIPK2 emerged as the most biologically and clinically relevant candidate. RIPK2 is a central kinase downstream of NOD1/2 signaling [22] and mediates activation of NF-κB [23] and MAPK [24] pathways following innate immune stimulation. Previous studies have implicated RIPK2 in several inflammatory and autoimmune disorders [25,26], whereas its relationship with cellular senescence in RA remains poorly understood. In the present study, RIPK2 was consistently upregulated in both the discovery and validation cohorts and showed robust diagnostic performance across datasets. Furthermore, elevated RIPK2 expression was confirmed in peripheral blood leukocytes from patients with RA. Importantly, silencing RIPK2 reduced LPS-induced expression of senescence markers and inflammatory cytokines while decreasing SA-β-gal-positive macrophages. These observations suggest that RIPK2 may contribute to senescence-associated inflammatory responses in macrophages. However, because our experiments were conducted in an in vitro LPS-induced macrophage model, these findings should be interpreted as evidence of association rather than definitive proof of a direct mechanistic pathway. Further studies are required to determine whether RIPK2 directly regulates senescence signaling or influences cellular senescence indirectly through inflammatory cascades.

### 3.3. Immune Infiltration and Molecular Subtypes

Immune infiltration analysis further supports the biological relevance of the hub genes identified in this study. All three hub genes displayed significant positive correlations with diverse immune cell subsets, and macrophages, monocytes as well as dendritic cells exhibited obvious correlational trends. Macrophages play a pivotal role in RA by producing pro-inflammatory cytokines [27], matrix-degrading enzymes, and mediators that promote synovial inflammation and joint destruction [28]. The strong association between RIPK2 and myeloid immune cells observed in this study, together with our experimental findings, raises the possibility that RIPK2 contributes to macrophage-mediated inflammatory amplification during RA. Considering that senescent macrophages acquire SASP characteristics capable of sustaining chronic inflammation, RIPK2 may represent one molecular link connecting innate immune activation with senescence-associated inflammatory remodeling in RA.

An additional strength of this study is the integration of multiple computational approaches to improve biomarker identification. Candidate genes were independently supported by differential expression analysis, WGCNA, and three machine learning algorithms, reducing the likelihood of selecting unstable biomarkers. Moreover, consensus clustering identified two molecular subtypes with distinct immune characteristics, suggesting that senescence-related molecular signatures may contribute to disease heterogeneity. Nevertheless, these computational findings should be regarded as hypothesis-generating, and their clinical utility requires validation in larger prospective cohorts before implementation in precision medicine.

### 3.4. Validation of RIPK2-Mediated Macrophage Senescence via the NF-κB Signaling Axis in Mouse Macrophages and Clinical Samples

To further confirm the biological relevance of the computational findings, we performed experimental validation at both the cellular and clinical levels. In LPS-stimulated RAW264.7 macrophages, RIPK2 knockdown significantly attenuated the upregulation of senescence-related genes (p16, p21, p53) and inflammatory cytokines (IL-6, TNF-α), reduced the secretion of TNF-α and IL-1β, and decreased SA-β-gal-positive cells. Accumulating review evidence has established that NF-κB acts as the master transcriptional switch governing SASP and cellular senescence across multiple cell types: genotoxic stress, oxidative damage and inflammatory stimuli converge to activate the IKK-NF-κB cascade, which directly transactivates the full panel of SASP pro-inflammatory mediators and cooperates with p53/p21 to amplify cell cycle arrest signals [29].

Specifically in macrophages, LPS-induced inflammatory stress robustly triggers NF-κB nuclear translocation; pharmacological inhibition of p65 phosphorylation or genetic ablation of IKKβ effectively suppresses macrophage senescence hallmarks including elevated p16/p21 and SA-β-gal staining, confirming NF-κB as an indispensable mediator of macrophage senescence-like inflammaging [30,31].

Mechanistically, RIPK2 is a well-characterized upstream signaling kinase positioned immediately upstream of the IKK-NF-κB axis downstream of NOD1/2 innate immune receptors [32]. Existing RAW264.7 macrophage studies further validate that RIPK2 overexpression amplifies NF-κB activation and subsequent senescence marker elevation upon LPS stimulation, while RIPK2 silencing blunts NF-κB-driven SASP and cell cycle inhibitory programs.

Consistent with this RIPK2–NF-κB signaling cascade, Western blot analysis further showed that LPS increased RIPK2, p16, and phosphorylated p65 (p-p65) protein levels, whereas RIPK2 knockdown inhibited these changes. The attenuation of p-p65 therefore supports a potential RIPK2–NF-κB axis that may contribute to macrophage senescence-like changes and inflammatory responses. In clinical samples, RIPK2 mRNA expression was significantly elevated in peripheral blood leukocytes from RA patients compared with healthy controls, and RIPK2 expression showed a positive correlation trend with plasma inflammatory factor levels. These independent lines of evidence support the potential involvement of RIPK2 in RA-associated inflammation and the macrophage senescence-like phenotype.

This study integrates bioinformatics analyses with experimental validation to identify cellular senescence-associated molecular signatures in RA and highlights RIPK2 as a candidate regulator of macrophage senescence-associated inflammatory responses. These findings expand our understanding of the interplay between cellular senescence and innate immunity in RA and provide a rationale for further investigation of RIPK2 as a potential biomarker and therapeutic target.

### 3.5. Limitations

Several limitations should be acknowledged. First, transcriptomic analyses relied primarily on publicly available datasets; although an independent external cohort was included for validation, additional multicenter validation with larger sample sizes is needed to improve the robustness and generalizability of the findings. Second, only RIPK2 was experimentally validated, whereas the biological functions of TNFAIP6 and SLC2A3 in RA remain to be investigated. Third, functional studies were limited to LPS-stimulated RAW264.7 macrophages and peripheral blood samples; moreover, the short-term LPS stimulation model cannot fully distinguish between transient inflammatory stress responses and bona fide cellular senescence, and the observed changes should therefore be interpreted as senescence-like rather than definitive senescence. Therefore, confirmation in primary human synovial macrophages and in vivo arthritis models is warranted to establish physiological relevance. Fourth, although RIPK2 knockdown attenuated LPS-induced p65 phosphorylation, suggesting potential involvement of the NF-κB pathway, the mechanistic evidence remains preliminary. Further experiments such as NF-κB inhibition, overexpression, or rescue assays are required to establish a definitive causal relationship between RIPK2 and macrophage senescence-like changes.

In addition, the clinical validation cohort was small (10 RA patients and 10 healthy controls), precluding robust adjustment for potential confounders such as age, sex, disease duration, disease activity, inflammatory markers, autoantibody status, and medication use. These factors may substantially influence inflammatory cytokine levels and RIPK2 expression; therefore, larger multicenter cohorts with comprehensive clinical phenotyping and multivariate analyses are needed. Furthermore, the discovery and external validation cohorts were derived from European populations, whereas the local clinical validation cohort consisted of Asian patients. Given the role of genetic background in RA susceptibility and progression, population-specific differences may affect the expression of senescence-related genes and inflammatory mediators; thus, the cross-ethnic generalizability of our findings should be interpreted with caution.

Additionally, the discovery cohort included RA patients with heterogeneous treatment backgrounds, including some treated with triple DMARD therapy, which may confound synovial gene expression and influence the identification of disease-related genes. Although external validation, machine learning-based feature selection, and independent experimental validation were performed to mitigate this bias, the influence of treatment cannot be fully excluded. The cohort was also restricted to established RA and normal synovium, excluding arthralgia, undifferentiated arthritis, and early RA to minimize diagnostic heterogeneity. While this improves specificity for established RA, it limits generalizability to earlier disease stages. Finally, RAW264.7 is a murine macrophage cell line that may not fully recapitulate human RA macrophage biology; validation in human primary macrophages or THP-1/U937 cells is warranted to confirm the role of RIPK2 in human macrophage senescence-like changes and RA pathogenesis.

## 4. Materials and Methods

### 4.1. Chemicals and Reagents

Lipopolysaccharide (LPS), reverse transcription kit, and RNAiMAX transfection reagent were purchased from Thermo Fisher Scientific (Waltham, MA, USA). The SYBR Green qPCR kit was obtained from Toyobo Co., Ltd. (Osaka, Japan). The BCA protein assay kit and senescence-associated β-galactosidase (SA-β-gal) staining kit were purchased from Beyotime Biotechnology (Shanghai, China). Anti-RIPK2, anti-p65, and anti-phospho-p65 (p-p65) antibodies were purchased from Proteintech Group (Wuhan, China). Anti-p16 antibody was obtained from HUABIO (Hangzhou, China). Anti-β-Actin antibody was purchased from Biosharp Biotechnology (Beijing, China). HRP-conjugated secondary antibodies were purchased from Beyotime Biotechnology (Shanghai, China). The following ELISA kits were used: Human IL-6 ELISA Kit (MultiSciences, Hangzhou, China; sensitivity: 0.37 pg/mL, dynamic range: 1.56–100 pg/mL), Human IL-1β ELISA Kit (MultiSciences, Hangzhou, China; sensitivity: 0.15 pg/mL, dynamic range: 3.91–250 pg/mL), Human IL-10 ELISA Kit (MultiSciences, Hangzhou, China; sensitivity: 0.43 pg/mL, dynamic range: 3.13–200 pg/mL), Mouse TNF-α ELISA Kit (MultiSciences, Hangzhou, China; sensitivity: 0.99 pg/mL, dynamic range: 10.94–700 pg/mL), and Mouse IL-1β ELISA Kit (MultiSciences, Hangzhou, China; sensitivity: 5.699 pg/mL, dynamic range: 23.44–1500 pg/mL). Other reagents and chemicals were of analytical grade and obtained from commercial sources. 

### 4.2. Data Collection and Preprocessing

The gene expression profile data used in this study were obtained from the GEO public database (https://www.ncbi.nlm.nih.gov/geo/, accessed on 18 March 2026). The GSE89408 dataset was selected for subsequent analysis. This dataset contains RNA-seq data generated on the Illumina HiSeq 2000 platform (GPL11154), with samples derived from synovial biopsy tissues of RA patients and healthy controls. The dataset contains a total of 218 synovial biopsy samples, including 152 samples from rheumatoid arthritis (RA) patients, 22 from osteoarthritis (OA) patients, 28 from healthy controls, 10 from patients with arthralgia, and 6 from patients with undifferentiated arthritis. The raw counts matrix was downloaded from the GEO database and normalized using the median of ratios method from the DESeq2 package. Low-expression genes were filtered out, and genes with count > 1 in at least 10% of samples were retained for subsequent differential expression analysis. All data processing was performed in the R environment.

### 4.3. Screening of Differentially Expressed Genes from Transcriptomic Datasets

Differential expression analysis between the RA and control groups was performed using the DESeq2 package (v1.46.0, Bioconductor Project, Boston, MA, USA). Differentially expressed genes (DEGs) were screened based on a negative binomial distribution model, with a threshold of *p*adj < 0.05 and |log_2_FC| > 0.5. A volcano plot was drawn to show the distribution of differential genes. Cellular senescence-related genes were collected from established senescence-associated resources and GeneCards (https://www.genecards.org, accessed on 18 March 2026), with GeneCards relevance score ≥ 7 used as an additional filtering criterion.

### 4.4. Construction of the Co-Expression Network

A gene co-expression network was constructed using filtered expressed genes/highly variable genes. Disease-associated genes were subsequently identified by intersecting WGCNA modules with DEGs and senescence-related genes [13]. The optimal soft threshold was selected according to scale-free network topological features, and power = 10 was chosen when signed R^2^ ≥ 0.85. The topological overlap matrix (TOM) was calculated, and co-expression modules were divided by hierarchical clustering. The minimum module size was set to 30. ME were calculated for each module, and their Pearson correlation with RA traits was analyzed to screen the key module with the most significant phenotypic association. GS was defined as the absolute value of the Pearson correlation coefficient between each gene expression profile and the RA phenotype, while MM was defined as the Pearson correlation coefficient between each gene expression profile and its corresponding module eigengene. The correlation between MM and GS was calculated using the Pearson product–moment correlation coefficient to verify the intra-module co-expression consistency and the strength of disease phenotype association for genes in the key module; statistical significance was determined by a two-sided Student’s *t*-test with *n* − 2 degrees of freedom.

### 4.5. Screening of Candidate Genes and Protein–Protein Interaction Network Analysis

The intersection of differentially expressed genes, cellular senescence-related genes, and WGCNA key module genes was taken, and the overlapping genes were defined as the candidate gene set. Candidate genes were imported into the STRING database (https://cn.string-db.org/, accessed on 18 March 2026) to construct a protein–protein interaction (PPI) network, and a confidence threshold was set to filter reliable interactions. The PPI network was visualized using the igraph package (v2.0.3, igraph core-development team), and topological parameters including node degree, betweenness centrality, and closeness centrality were calculated [33]. The importance of genes in the network was evaluated based on node degree to screen potential hub genes.

### 4.6. Machine Learning

To further screen key diagnostic biomarkers for RA, three machine learning methods were used for feature selection of candidate genes. The “glmnet” package (v4.1-10, original developers Trevor Hastie, Jerome Friedman, Robert Tibshirani, Stanford University, Stanford, California, USA) in R was used to perform LASSO regression, which aims to select linear models by shrinking coefficients of less important variables to zero, thereby retaining the most relevant features for predictive modeling. The optimal regularization parameter λ (λ.min) was selected through ten-fold cross-validation, and genes with non-zero coefficients were retained as feature genes [34]. Random forest was used to construct a classification model using the randomForest package (v4.7-1.2, original authors Leo Breiman, Berkeley, CA, USA), with ntree = 500 and mtry set as the square root of the number of features. Gene feature importance was evaluated based on the mean decrease in Gini index, and model stability was assessed through ten-fold cross-validation. SVM-RFE was implemented using the e1071 package (v1.7-16, TU Wien/CRAN community, Vienna, Austria) with a radial basis kernel function. The optimal feature subset was screened through recursive feature elimination, and the feature subset size was optimized through ten-fold cross-validation. The feature genes screened by the three methods were intersected to obtain the final core hub gene set, and a circular diagram of chromosomal position distribution was drawn [35].

### 4.7. Construction and Performance Evaluation of the Diagnostic Model

Based on the core hub genes, a nomogram prediction model was constructed using the rms package (v6.8-2, original developer Frank E. Harrell Jr., Nashville, TN, USA). Multivariate Logistic regression analysis was performed to calculate regression coefficients and assign scores for each gene. The scores of individual genes were summed to obtain a total score, which was converted to RA disease probability for intuitive prediction of individual risk. Multiple methods were used to comprehensively evaluate model performance: ROC curves were drawn and AUC was calculated to evaluate diagnostic discrimination; calibration curves were plotted to evaluate prediction accuracy; decision curve analysis (DCA) was performed to evaluate potential clinical relevance.

### 4.8. Expression Analysis and Diagnostic Efficacy Evaluation of Core Genes

To verify the expression differences and diagnostic value of core genes in RA, the expression levels of the three core hub genes were first examined in the training cohort GSE89408, and further validated in the independent external validation cohort GSE55457. Violin plots were used to visualize the expression differences in core genes between the RA and control groups, and the Wilcoxon rank-sum test was applied to assess the statistical significance of expression differences between the two groups in both cohorts.

Receiver operating characteristic (ROC) curves were further constructed to evaluate the diagnostic efficacy of individual core genes for RA. Diagnostic indicators including the area under the curve (AUC), optimal cutoff value, sensitivity, specificity, positive predictive value, negative predictive value, positive likelihood ratio, and negative likelihood ratio were calculated in both cohorts, to systematically evaluate the potential of core genes as diagnostic biomarkers for RA and verify the robustness of the findings.

### 4.9. Immune Infiltration and Immune Pathway Enrichment Analysis

ssGSEA was used to calculate enrichment scores of 28 immune cell types in each sample to assess the relative abundance of immune cells in the RA immune microenvironment. Differences in enrichment scores of immune cells and 13 immune-related pathways between the RA and control groups were compared, and immune infiltration profile differences were visualized using heatmaps and violin plots. Correlations between core hub gene expression and enrichment scores of immune cells and immune pathways were further analyzed, and correlation heatmaps were drawn to show the association strength. Correlations among the 28 immune cell types were also analyzed to reveal the synergistic relationships among immune cell subsets. Gene Set Variation Analysis (GSVA) was conducted on C7 immunologic signatures and hallmark gene sets downloaded from the MSigDB database (https://www.gsea-msigdb.org/gsea/msigdb, accessed on 18 March 2026) to characterize multi-dimensional pathway enrichment landscapes of the RA immune microenvironment.

### 4.10. Regulatory Network Construction and Molecular Subtyping

To characterize the upstream regulatory mechanisms of core genes, miRNAs and transcription factors targeting core genes were retrieved through the NetworkAnalyst platform (https://www.networkanalyst.ca, accessed on 18 March 2026), and a multi-level miRNA-TF-mRNA regulatory network was constructed.

Based on the expression profiles of the three core hub genes, consensus clustering analysis was performed using the ConsensusClusterPlus package (v1.66.0, original developers Matt Wilkerson, Peter Waltman, Bioconductor Project, Boston, MA, USA) to divide RA patients into different molecular subtypes. The optimal number of clusters was determined by cumulative distribution function (CDF) curves, consensus heatmaps, and cluster scores. Heatmaps and box plots were used to show expression differences in core genes between the two subtypes, and the validity of subtype classification was verified by principal component analysis (PCA). Differentially expressed genes between the two subtypes were screened, and the top 10 differentially expressed genes were selected for display. GSEA enrichment analysis was performed on differentially expressed genes between subtypes to compare enrichment differences at the pathway level. Meanwhile, ssGSEA was used to assess immune cell enrichment abundance in each subtype, and the immune phenotypic characteristics of different subtypes were compared.

### 4.11. Prediction of Potential Targeted Drugs and Molecular Docking Validation

Small-molecule compounds and drugs interacting with core hub genes were retrieved through the CTD (https://ctdbase.org, accessed on 18 March 2026) and CMap (https://clue.io/cmap, accessed on 18 March 2026) databases to screen potential therapeutic drugs. A drug–gene interaction network was constructed for visualization.

To further verify the binding ability of drugs to target proteins, representative drug–protein combinations were selected for molecular docking analysis. Three-dimensional structures of proteins were obtained from the PDB or UniProt database, and three-dimensional structures of small-molecule drugs were obtained from the PubChem database. Molecular docking was performed using AutoDock Vina software (v1.2.5, Center for Computational Structural Biology, Scripps Research Institute, La Jolla, CA, USA) to calculate protein–ligand binding energy. The binding mode of small drug molecules to the protein active pocket and interactions between key amino acid residues were analyzed to verify the binding feasibility of potential drugs to target proteins from a structural biology perspective.

### 4.12. Clinical Sample Collection

Peripheral blood samples were collected from 10 RA patients and 10 age- and sex-matched healthy controls. Peripheral blood leukocytes and plasma were isolated. All RA patients met the 2010 ACR/EULAR classification criteria for RA. All participants were informed that their clinical data and blood would be used for research, and signed informed consent. All experiments involving human blood samples followed the guidelines of the Declaration of Helsinki and were approved by the Institutional Ethics Research Committee of Yijishan Hospital (approval number: 2022-20). All subjects signed informed consent forms.

### 4.13. Cell Culture and Treatment

The mouse macrophage cell line RAW264.7 was cultured in high-glucose DMEM medium containing 10% fetal bovine serum in a 37 °C, 5% CO_2_ incubator with saturated humidity. A macrophage inflammation and senescence model was induced by lipopolysaccharide (LPS) at a final concentration of 30 ng/mL for 24 h.

### 4.14. siRNA Transfection

Small interfering RNAs targeting RIPK2 (siRIPK2) and negative control siRNA (siNC) were designed and synthesized by Sangon Biotech. All siRNA sequences are listed in Supplementary Files, and all Supplementary Materials are compiled into a single Word document. Cell transfection was performed using RNAiMAX transfection reagent according to the manufacturer’s instructions. After 48 h of transfection, knockdown efficiency was verified by Western blot, and the siRNA sequence with the best knockdown effect was selected for subsequent functional experiments.

### 4.15. Quantitative Real-Time PCR (qPCR)

Total RNA was extracted from cells and clinical samples using Trizol reagent. After detecting RNA concentration and purity, cDNA was synthesized by reverse transcription. Quantitative real-time PCR was performed using the SYBR Green method with ACTB as the internal reference gene. All primer sequences for qPCR are listed in Supplementary Files, and all Supplementary Materials are compiled into a single Word document. Relative gene expression was calculated using the 2^−ΔΔCt^ method. Detected genes included RIPK2, P16, P21, P53, IL-6, and TNF-α.

### 4.16. Enzyme-Linked Immunosorbent Assay (ELISA)

The concentrations of inflammatory factors in plasma and cell supernatants, including TNF-α, IL-6, IL-1β, and IL-10, were detected using ELISA kits. All operations were performed strictly according to the kit instructions. The absorbance value of each well was measured by a microplate reader, and the concentration of each cytokine in the samples was calculated according to the standard curve.

### 4.17. Western Blot

Total cellular protein was extracted, and protein concentration was determined by the BCA method. Equal amounts of protein were separated by SDS-PAGE electrophoresis and electrotransferred to PVDF membranes. After blocking with 5% skim milk at room temperature for 1 h, primary antibodies were added and incubated overnight at 4 °C. After washing with TBST, HRP-labeled secondary antibodies were added and incubated at room temperature for 1 h. ECL chemiluminescent reagent was used for development, and images were acquired by a gel imaging system. β-Actin was used as the internal reference, and grayscale quantitative analysis was performed using ImageJ software (v1.54g, National Institutes of Health, Bethesda, MD, USA).

### 4.18. β-Galactosidase Staining

The senescence-associated β-galactosidase (SA-β-gal) staining kit was used to detect the cellular senescence phenotype. All operations were performed according to the kit instructions. After washing cells with PBS, cells were fixed with 4% paraformaldehyde, and staining working solution was added, followed by incubation at 37 °C overnight. Senescent-positive cells stained blue were observed and counted under an optical microscope, and the proportion of senescent cells was calculated.

### 4.19. Statistical Analysis

Statistical analyses were performed using R software (v4.4.1, R Core Team, Vienna, Austria) and GraphPad Prism 9.0 (v9.0.0, GraphPad Software, San Diego, CA, USA). Normality was assessed by the Shapiro–Wilk test, and variance homogeneity by Levene’s test. For two-group comparisons, unpaired two-tailed Student’s *t*-test was used when data were normally distributed with equal variances; otherwise, the Mann–Whitney U test was applied. For multiple comparisons, one-way ANOVA followed by Tukey’s post hoc test was used for parametric data, and the Kruskal–Wallis test followed by Dunn’s post hoc test was used for nonparametric data. Data are presented as mean ± SD from at least three independent biological replicates unless otherwise stated; *n* represents biological replicates for in vitro experiments and independent patient samples for clinical analyses. For box plots with individual data points, boxes represent the median and interquartile range (IQR), whiskers extend to 1.5× IQR, and individual dots represent independent patient samples. Correlation was assessed by Pearson’s correlation for normally distributed data or Spearman’s rank correlation for non-normally distributed data. All statistical tests were two-tailed, and *p* < 0.05 was considered statistically significant.

## 5. Conclusions

In conclusion, this study integrated multiple bioinformatics approaches and experimental validation to identify three cellular senescence-related hub genes (TNFAIP6, SLC2A3, and RIPK2) in RA. Among them, RIPK2 was selected for further functional validation due to its prominent diagnostic performance, strong correlation with immune senescence pathways, and central position in regulatory networks. We demonstrated that RIPK2 is significantly upregulated in RA and promotes LPS-induced macrophage senescence-like phenotype and inflammatory responses, while RIPK2 knockdown effectively attenuates these effects. These findings not only provide new insights into the pathogenesis of RA from the perspective of cellular senescence, but also suggest RIPK2 as a promising diagnostic biomarker and therapeutic target for RA. Further studies with larger sample sizes and in vivo models are warranted to validate the clinical translational value of RIPK2 in RA.

## Data Availability

All data generated or analyzed during this study are included in this article and its Supplementary Information files. Other relevant information is available from the corresponding author upon reasonable request.

## Supplementary Materials

The following supporting information can be downloaded at: https://www.mdpi.com/article/10.3390/ijms27167493/s1.
