# Supplementary material for: Identification of Cellular Senescence-Related Hub Genes in Rheumatoid Arthritis from Bioinformatics Analysis Through Machine Learning up to Verifications in Mouse Macrophages and Tests in Patients
Source: Int J Mol Sci. 2026 Aug 21;27(16):7493. doi: 10.3390/ijms27167493 (PMC13513530; doi:10.3390/ijms27167493)
Supplement: Supplementary file 1 [file ijms-27-07493-s001.zip › ijms-4484832-supplementary.pdf]

## Sequences of the gene-specific primers and siRNA used in this study

siRNA Sequences

| | |
|---|---|
| siRIPK2-1 -s | GACCUAAAGACUCAGAAUA/dT//dT/ |
| siRIPK2-1 -a | UAUUCUGAGUCUUUAGGUC/dT//dT/ |
| siRIPK2-2 -s | GGAGGAACAAUCAUCUAUA/dT//dT/ |
| siRIPK2-2 -a | UAUAGAUGAUUGUUCCUCC/dT//dT/ |
| siRIPK2-3 -s | CUCAAGUACUAUUCACUUA/dT//dT/ |
| siRIPK2-3-a | UAAGUGAAUAGUACUUGAG/dT//dT/ |

Mouse Primers

| | |
|---|---|
| RIPK2-FORWARD | CTCGTGTTCCTTGGCTGTAATAAGTC |
| RIPK2-REVERSE | TCATCTGGCTCACAATGGCTTCC |
| ACTB-FORWARD | GATGGTGGGAATGGGTCAGAAGG |
| ACTB-REVERSE | TTGTAGAAGGTGTGGTGCCAGATC |
| p16-FORWARD | TTCCGCTGGGTGCTCTTTGTG |
| p16-REVERSE | GAAGCTATGCCCGTCGGTCTG |
| p21-FORWARD | CGAGAACGGTGGAACTTTGACTTC |
| p21-REVERSE | CGGGACCCAGGGCTCAGG |
| p53-FORWARD | CGCCGACCTATCCTTACCATCATC |
| p53-REVERSE | CTCCCAGGGCAGGCACAAAC |
| TNFα-FORWARD | ACGCTCTTCTGTCTACTGAACTTCG |
| TNFα-REVERSE | TGGTTTGTGAGTGTGAGGGTCTG |
| IL-6-FORWARD | TTCTTGGGACTGATGC |
| IL-6-REVERSE | GTGGTATCCTCTGTGAAGTCTCCTC |

Human Primers

| | |
|---|---|
| RIPK2-FORWARD | CTGGATCTCAAAGGGCTGCA |
| RIPK2-REVERSE | TTGCTCTGGATCCACTGCTG |
| ACTB-FORWARD | ATTGCCGACAGGATGCAGAA |
| ACTB-REVERSE | CGGACTCGTCATACTCCTGC |
| p16-FORWARD | GCCCAACGCACCGAATAGTTAC |
| p16-REVERSE | GCAGCAGCTCCGCCACTC |
| p21-FORWARD | ACCTGTCACTGTCTTGTACCCTTG |
| p21-REVERSE | GCGTTTGGAGTGGTAGAAATCTGTC |
| p53-FORWARD | CCTCCTCAGCATCTTATCCGAGTG |
| p53-REVERSE | CCAACCTCAGGCGGCTCATAG |
| TNFα-FORWARD | CTTCCAGCTGGAGAAGGGTG |
| TNFα-REVERSE | CCCAAAGTAGACCTGCCCAG |
| IL-6-FORWARD | AGTGAGGAACAAGCCAGAGC |
| IL-6-REVERSE | GGTCAGGGGTGGTTATTGCA |

# Figure legend

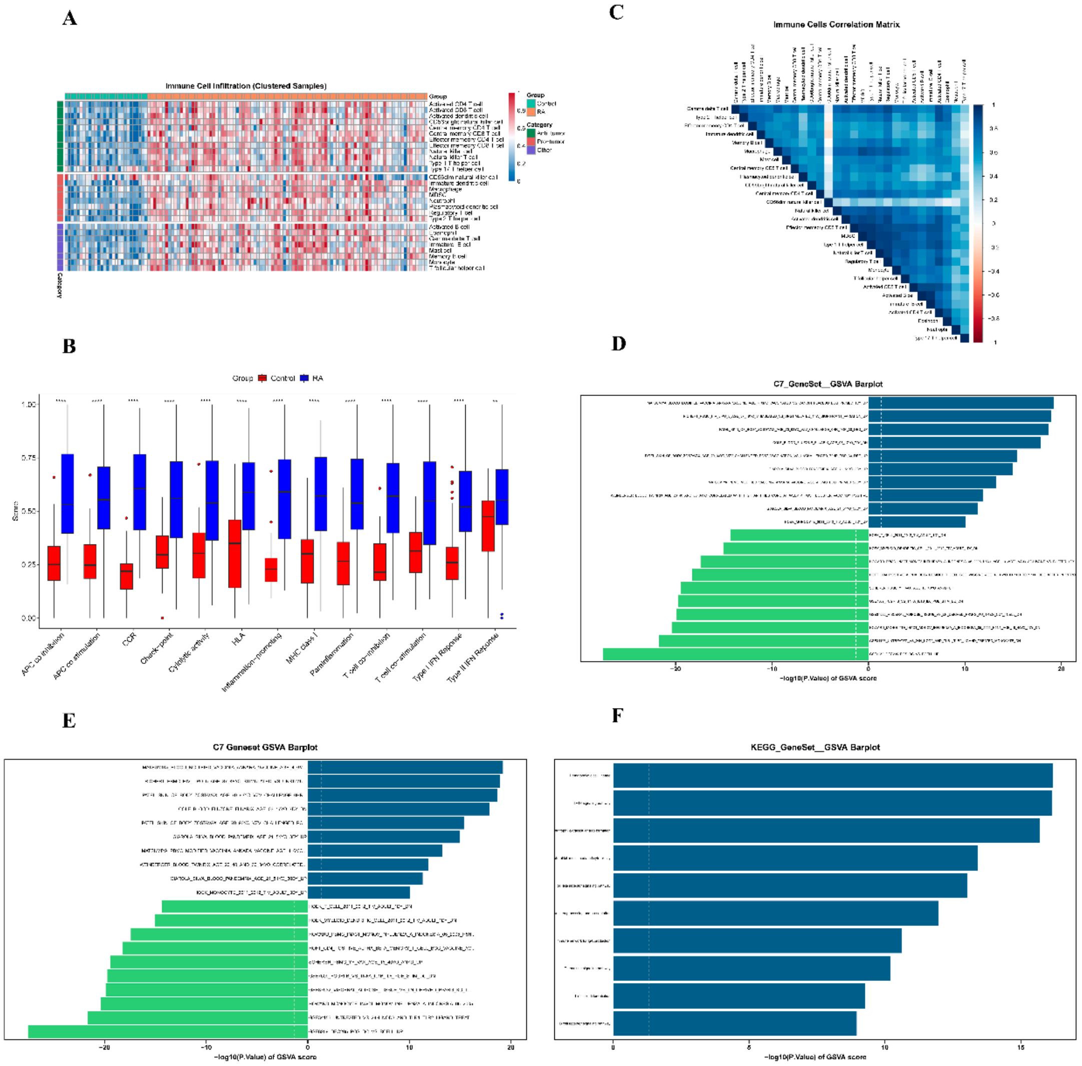


Supplementary Figure S1. Immune infiltration and pathway enrichment analysis of rheumatoid arthritis. (A) Heatmap of enrichment scores for 28 immune cell types. Samples are clustered by group, showing obvious differences in immune infiltration profiles between the RA and control groups. (B) Box plots of immune cell/pathway enrichment scores. Blue represents the RA group, red represents the control group. The enrichment scores of multiple immune cells and pathways are significantly higher in the RA group than in the control group. (C) Correlation matrix heatmap of immune cells, showing positive correlations among various immune cell subsets. (D-E) Bar plots of GSVA enrichment analysis of C7 immune-related gene sets, showing differentially enriched immune gene sets ranked by enrichment significance. (F) Bar plot of GSVA enrichment analysis of KEGG pathways, showing significantly enriched KEGG signaling pathways in RA.

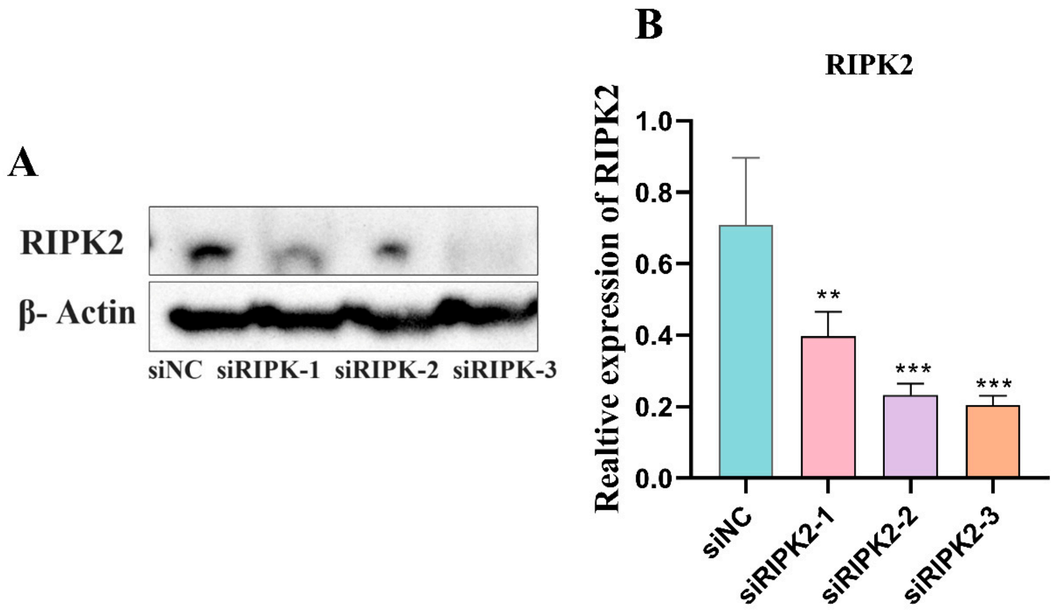


Supplementary Figure S2. Validation of knockdown efficiency of three independent RIPK2 siRNAs
(A) Western blot analysis of RIPK2 protein expression in cells transfected with siNC, siRIPK2-1, siRIPK2-2 and siRIPK2-3, with β-Actin served as the internal reference.
(B) Quantitative statistical analysis of relative RIPK2 protein expression. $**P < 0.01$, $***P < 0.001$ compared with the siNC group. siNC, negative control siRNA.

**Supplementary Table S1. Demographic and clinical characteristics of RA patients and healthy controls.**

| | **RA Patients** | **Healthy controls** |
|---|---|---|
| Patients, *n* | 10 | 10 |
| Age, years | 61.50 ± 11.10 | 61.60 ± 11.14 |
| Female/male, no.s | 6/4 | 6/4 |
| Disease duration (years) | 4.90 ± 1.79 | N.A |
| DAS28-ESR | 3.57±0.91 | N.A |
| CRP (mg/L) | 19.72 ± 21.74 | N.A |
| ESR (mm/h) | 38.30 ± 30.59 | N.A |
| RF (IU/mL) | 259.85 ± 375.49 | N.A |
| Anti-CCP titer (U/mL) | 212.66 ± 212.83 | N.A |
| Previous medications, n (%) | | |
| Glucocorticoid use, n (%) | 6 (60%) | 0 (0%) |
| csDMARD use (MTX/SASP/HCQ), n (%) | 9 (90%) | 0 (0%) |
| bDMARD / targeted-DMARD, n (%) | 2 (20%) | 0 (0%) |
